# RosBREED: bridging the chasm between discovery and application to enable DNA-informed breeding in rosaceous crops

**DOI:** 10.1038/s41438-020-00398-7

**Published:** 2020-11-01

**Authors:** Amy F. Iezzoni, Jim McFerson, James Luby, Ksenija Gasic, Vance Whitaker, Nahla Bassil, Chengyan Yue, Karina Gallardo, Vicki McCracken, Michael Coe, Craig Hardner, Jason D. Zurn, Stan Hokanson, Eric van de Weg, Sook Jung, Dorrie Main, Cassia da Silva Linge, Stijn Vanderzande, Thomas M. Davis, Lise L. Mahoney, Chad Finn, Cameron Peace

**Affiliations:** 1 Michigan State University, East Lansing, MI 48824 USA; 2 Washington State University, Wenatchee, WA 98801 USA; 3 University of Minnesota, St. Paul, MN 55108 USA; 4 Clemson University, Clemson, SC 29634 USA; 5 University of Florida, Wimauma, FL 33598 USA; 6 USDA-ARS, Corvallis, OR 97333 USA; 7 Washington State University, Puyallup, WA 98371 USA; 8 Washington State University, Pullman, WA 99164 USA; 9 Cedar Lake Research Group, Portland, OR 97215 USA; 10 University Queensland, Brisbane, QLD Australia; 11 University of Minnesota, St. Paul, MN 55108 USA; 12 Wageningen University and Research, 6700 AA Wageningen, The Netherlands; 13 Washington State University, Pullman, WA 99164 USA; 14 University of New Hampshire, Durham, NH 03824 USA

**Keywords:** Plant breeding, Plant breeding

## Abstract

The Rosaceae crop family (including almond, apple, apricot, blackberry, peach, pear, plum, raspberry, rose, strawberry, sweet cherry, and sour cherry) provides vital contributions to human well-being and is economically significant across the U.S. In 2003, industry stakeholder initiatives prioritized the utilization of genomics, genetics, and breeding to develop new cultivars exhibiting both disease resistance and superior horticultural quality. However, rosaceous crop breeders lacked certain knowledge and tools to fully implement DNA-informed breeding—a “chasm” existed between existing genomics and genetic information and the application of this knowledge in breeding. The RosBREED project (“Ros” signifying a Rosaceae genomics, genetics, and breeding community initiative, and “BREED”, indicating the core focus on breeding programs), addressed this challenge through a comprehensive and coordinated 10-year effort funded by the USDA-NIFA Specialty Crop Research Initiative. RosBREED was designed to enable the routine application of modern genomics and genetics technologies in U.S. rosaceous crop breeding programs, thereby enhancing their efficiency and effectiveness in delivering cultivars with producer-required disease resistances and market-essential horticultural quality. This review presents a synopsis of the approach, deliverables, and impacts of RosBREED, highlighting synergistic global collaborations and future needs. Enabling technologies and tools developed are described, including genome-wide scanning platforms and DNA diagnostic tests. Examples of DNA-informed breeding use by project participants are presented for all breeding stages, including pre-breeding for disease resistance, parental and seedling selection, and elite selection advancement. The chasm is now bridged, accelerating rosaceous crop genetic improvement.

## This review

Rosaceous fruit, nut, and floral crops provide high-value nutritious foods, contribute to our esthetic enjoyment, and are economically important globally. Related through their ancestral genome^1^, rosaceous crops have been selected and bred to provide an assortment of superior cultivars on which modern production is based. However, the next generation of cultivars is needed to improve consumer satisfaction, profitability for industry stakeholders, and environmental sustainability. In the “genomics era”, many crop scientists routinely access database resources, leverage increasingly detailed knowledge of plant genomes, and apply genetic tools to significantly enhance the efficiency and effectiveness of new-cultivar development. However, adoption of such DNA-informed breeding had lagged in rosaceous crops, with a daunting chasm between ongoing scientific discoveries and practical breeding applications. The RosBREED projects were a 10-year collaborative effort that bridged this chasm. This review presents a synopsis of the history, approach, deliverables, and impacts of RosBREED, highlighting synergistic global collaborations and future needs.

## RosBREED in a historical context

An extraordinary coming-together of the global rosaceous research community^2^ coincided with the U.S. crop industry’s prioritization of new-cultivar development as a research goal^3^. Equally important, the USDA-NIFA Specialty Crop Research Initiative (SCRI) provided significant funding opportunities and stimulated investment in breeding. RosBREED was an outcome of this unprecedented situation. The RosBREED approach was based on the premise that diverse rosaceous crops all shared a need for genomics information and diagnostic tools and envisioned addressing this need by a multi-crop, multi-state, transdisciplinary research and extension effort. Rosaceous crop breeders, industries, and allied scientists, united in this common goal, were the foundation upon which the RosBREED projects were developed.

### The Rosaceae family

Crops in the Rosaceae family are produced worldwide, primarily in temperate climates. While most are grown for their fruit, in both fresh and processed forms, the family also includes important nut (e.g., almond) and ornamental (e.g., rose) crops. The fruit crops, comprising apple, apricot, blackberry, nectarine, peach, pear, plum, red and black raspberry, strawberry, sweet cherry, and sour cherry, exhibit wide morphological diversity for fruit characteristics and plant growth habit. These fruit, nut, and ornamental crops are placed within two Rosaceae subfamilies: (1) Rosoideae, including *Fragaria* (strawberry), *Rubus* (caneberries), and *Rosa* (rose); and (2) Spiraeoideae, comprising the Pyreae tribe [*Malus* (apple) and *Pyrus* (pear)] and the Amygdaleae tribe [*Prunus* stone fruit and nut crops (almond, apricot, peach, nectarine, plum, sweet cherry, and sour cherry)]^4^. The genome of the ancestral Rosaceae progenitor is currently understood to have had nine chromosomes (*x* = 9). Synteny analyses^1^ describe the large structural changes hypothesized to have produced a range of base chromosome numbers: Rosoideae (*x* = 7), *Prunus* (*x* = 8), and *Malus*/*Pyrus* (*x* = 17). Apple and pear behave as diploids but are considered to be ancient polyploids^5,6^. The Rosaceae family also includes tetraploids (sour cherry and some apple, plum, blackberry, and rose species), hexaploids (some plum and blackberry species), octoploids (cultivated strawberry and some blackberry species), and higher ploidy crops (blackberry). Most crop members are highly heterozygous and some are obligate outcrossers exhibiting a gametophytic self-incompatibility system (apple, pear, most *Prunus* species). In commercial fruit tree production, all nursery trees consist of scions grafted on rootstocks, the latter usually being wild related species or intentionally bred interspecific hybrids.

### U.S. Rosaceae industry

Rosaceous crops in the U.S. are grown on more than 700,000 hectares, with a total production of 10 million tonnes and a value of utilized production of more than $13.5 billion (Table 1). In the U.S., most are produced on the west and east coasts and around the Great Lakes, as these regions experience moderate winter and spring temperatures and generally have sufficient water availability, either as rainfall or irrigation. However, local producers of tree fruit, berry, and ornamental crops are important throughout the country. Domestic production and delivery to consumers are enabled by a nationwide supply chain infrastructure of market intermediaries (packers, processors, brokers, and shippers). Almond, apple, cherry, pear, and strawberry are also valuable export crops.

**Table 1:** Rosaceous crops grown in the U.S. in 2018: bearing hectares, total production, and value of utilized production in 2018^193,194^.

Crop	Bearing hectares	Total production (metric tonnes equivalent)	Value of utilized production (US$1000)
Almond	441,100	1,698,700	5,468,040
Apple	117,800	4,652,500	3,013,713
Apricot	4,300	35,900	48,465
Blackberry	2,600	18,300	20,100
Cherry, sweet	34,400	312,400	637,700
Cherry, sour	14,200	135,300	56,635
Nectarine	5,700	109,300	119,650
Peach	30,100	591,000	511,226
Pear	18,700	730,700	428,940
Plum	5,700	90,700	92,570
Prune	17,800	253,700	194,832
Raspberry	6,800	99,200	367,001
Strawberry	19,900	1,296,300	2,670,523
Rose	–	–	28,069
Total	719,100	10,025,000	13,654,464

For the rose, only the value of utilized production was available. For blackberry, figures were only available for 2017^195^

In 2003, challenged by globalization of trade and foreign competition, industry stakeholders in rosaceous crops across the country developed a comprehensive, proactive strategy to address production and marketing challenges and increase profitability. As documented in the National Tree Fruit Technology Roadmap^3^, they envisioned a systematic reduction in per unit production costs by addressing production limitations and improving quality of the product delivered to consumers. New-cultivar development, undertaken with increased efficiency enabled by the routine adoption of DNA-informed breeding, was identified as a major research need.

### Rosaceae crop breeding

Until the 1900s, when scientifically based U.S. crop breeding programs commenced, rosaceous crop producers grew scion and rootstock cultivars mostly derived from chance seedlings from European selections. Subsequently, U.S. breeders have made considerable progress utilizing germplasm native to North America or other centers of diversity. Certain accessions of crop wild relatives have been effectively used as sources of desirable attributes such as disease resistance. Targeted collection expeditions to global centers of domestication have markedly increased diversity in U.S. germplasm collections and breeding programs (e.g., apple^7^, strawberry^8^, cherry^9^). However, the principal cultivars currently in commercial production, and even most breeding program material, typically can be traced to a limited number of founder cultivars. Another constraint on development of new superior cultivars has been the relatively limited number of U.S. breeding programs. At present, there are 100 active public and private sector Rosaceae crop breeding programs in the U.S., led by 53 breeders, many of whom work on more than one crop (M. Coe & C. Peace, pers. comm.). Most private programs are in California while public programs are generally in states with significant production, hosted by land-grant universities or the USDA.

Programs typically focus on improved fruit, nut, and/or floral quality and other high-impact stakeholder-driven production traits such as disease resistance and flowering/fruiting traits related to productivity. However, until recently, breeders had little understanding of the genetic control of these traits. A typical breeding strategy was a pedigree approach in which breeding populations based on biparental crosses were generated based on empirical experience of the phenotypes and an impression of general combining abilities of parents. In most cases, an inherently high level of heterozygosity resulted in a wide range of phenotypic variation among offspring, providing ample opportunities for selection. High heterozygosity, low heritability, and polygenic control for traits such as fruit sweetness complicated the breeder’s ability to identify the best parents and predict the best parental combinations, a challenge exacerbated in polyploids. Acquisition of experimental knowledge was further limited, especially in tree crops, by extended juvenility (with generation times of 2–7 years), large individual plant sizes, and the need for extensive field testing. As in other crops, breeders assumed available information on pedigrees of cultivars and other materials in their programs was correct, but genetic identity errors often reduced the ability to effectively design crosses that achieve trait targets and widen the genetic base.

Breeding for disease resistance is particularly challenging for rosaceous crops. Disease resistance alleles are often present in exotic germplasm (crop wild relatives and unadapted material) but rarely in elite modern cultivars. Resistance sources almost exclusively have numerous highly undesirable horticultural attributes, such as fruit that are small, bitter, astringent, and with poor handling and storage qualities. Eliminating undesirable attributes from exotic sources while introducing even a single resistance allele can take many generations. With each generation requiring multiple years and considerable space and cost, the challenge is often considered intractable for a given breeding program. Traditional backcrossing to introgress resistance alleles is not feasible due to the high heterozygosity of rosaceous crops. Furthermore, in most rosaceous crops, the presence of multiple resistance alleles for a disease is impossible to confirm by disease screening because the presence of a single resistance allele can phenotypically mask the presence of additional alleles; therefore, DNA markers for the resistance alleles are needed. Given such challenges, it is no surprise very few commercially competitive cultivars of rosaceous crops are disease-resistant.

### “The Chasm” and stakeholder needs addressed

In 2009, scientific advances in molecular genetics and genomics were accelerating across crop species. As for many other agriculturally important organisms, a biological/bioinformatics revolution was underway in rosaceous crops. Low-density genetic linkage maps were available for most crops. The apple^5^, peach^10^, and diploid strawberry genomes^11^ were being sequenced in their entirety. The Genome Database for Rosaceae (GDR) [https://www.rosaceae.org/]^12^, initiated in 2003, emerged as the international standard. More than 250 major-effect loci (Mendelian trait loci = MTLs), quantitative trait loci (QTLs), and sometimes identities and sequences of their underlying genes, were reported^13^. These genomics resources provided exciting opportunities for practical application in breeding programs, such as the *S* locus, with its self-compatible vs. self-incompatible alleles in cherry and almond.

However, for only a handful of important traits were such DNA-based diagnostic tools available. In fact, a huge chasm gaped between the acquired genetics and genomics knowledge and practical application in rosaceous crop breeding^14^. This situation occurred despite the tremendous potential utility of molecular genetics and genomics information and commonly stated promises it would increase breeding efficiency, especially for crops with long generation times and for traits requiring extensive field space and expensive phenotyping. Distressingly, while stakeholders placed high priority on new disease-resistant cultivars, few such cultivars were commercially relevant. Choice of parents and progeny selection based on phenotype alone could bring in some desirable disease resistance attributes but did not efficiently enable purging of undesirable “wild” chromosomal segments that often imparted unacceptable horticultural quality. Producers were understandably frustrated that breeders were unable to utilize emerging genomics technologies to combine desirable horticultural quality and disease resistance in new cultivars.

Despite the obvious benefits of DNA-informed breeding, rosaceous crop breeders faced numerous barriers discouraging its routine use in their programs. Often, they lacked the confidence to utilize exotic germplasm and to base selection in segregating progeny on putative genotype rather than observable phenotype. They were also concerned about implementing such marker-assisted selection (MAS) where linkage relationships among alleles were not defined between trait loci linked on the same chromosomes. Costs were considered prohibitive for genotyping thousands of seedlings. Finally, most programs lacked personnel trained in the implementation of DNA-informed breeding, and, as in other specialty crops, had little access to high-throughput public or private sector diagnostic services. Thus, rosaceous crop breeding programs shared a number of critical needs to: (1) validate and newly discover trait loci in breeding-relevant germplasm, (2) define linkage relationships, (3) design diagnostic genetic markers, (4) identify cost-efficient strategies, (5) develop competencies in necessary diagnostics technologies, and (6) identify diagnostics service providers^15^.

The two consecutive RosBREED projects from 2009 to 2019 were explicitly designed to address those needs and bridge the chasm between genetic discovery and sustainable implementation of DNA-informed rosaceous crop breeding. Achieving the project objectives would enable U.S. programs to routinely apply molecular genetics tools to efficiently, accurately, and rapidly breed new cultivars improved for stakeholders’ priority traits. The first project (2009–2013), “RosBREED: Enabling marker-assisted breeding in Rosaceae”, abbreviated as “RosBREED 1”, focused on fruit quality in apple, peach, strawberry, and sweet and sour cherry^16^. These crops and fruit quality were chosen because industry stakeholders supported DNA-informed breeding as a high research priority and believed enhanced fruit quality could improve consumer demand and their own profitability. The second project (2014–2019), “RosBREED: Combining disease resistance and horticultural quality in new rosaceous cultivars”, abbreviated as “RosBREED 2”, added blackberry, pear, rose, and *Prunus* rootstocks with an additional target trait of disease resistances prioritized by stakeholders^17^.

RosBREED 1 and RosBREED 2 were supported by the USDA-NIFA Specialty Crop Research Initiative (SCRI) program, an unprecedented federal grant program initiated in 2008 to support transdisciplinary research and extension activities addressing industry needs in specialty crops. In fact, the SCRI arose in response to stakeholder efforts such as the National Tree Fruit Technology Roadmap. Another key partner was the U.S. Rosaceae Genomics, Genetics, and Breeding Executive Committee (RosEXEC). Formed in 2005 and broadly representing industry, research, and extension interests among U.S. rosaceous crops, RosEXEC helps identify and communicate both stakeholder priorities and research community needs (https://www.rosaceae.org/community/us_rosexec). In 2011, the European Union funded a 4.5-year project designed to address the same chasm targeted in RosBREED, entitled “FruitBreedomics – Integrated approach for increasing breeding efficiency in fruit tree crops”. This project included only two crops, apple and peach, but the synergy among scientists in RosBREED and FruitBreedomics accelerated and enhanced the contributions from both projects^18^.

### The RosBREED approach

**Fig. 1:**
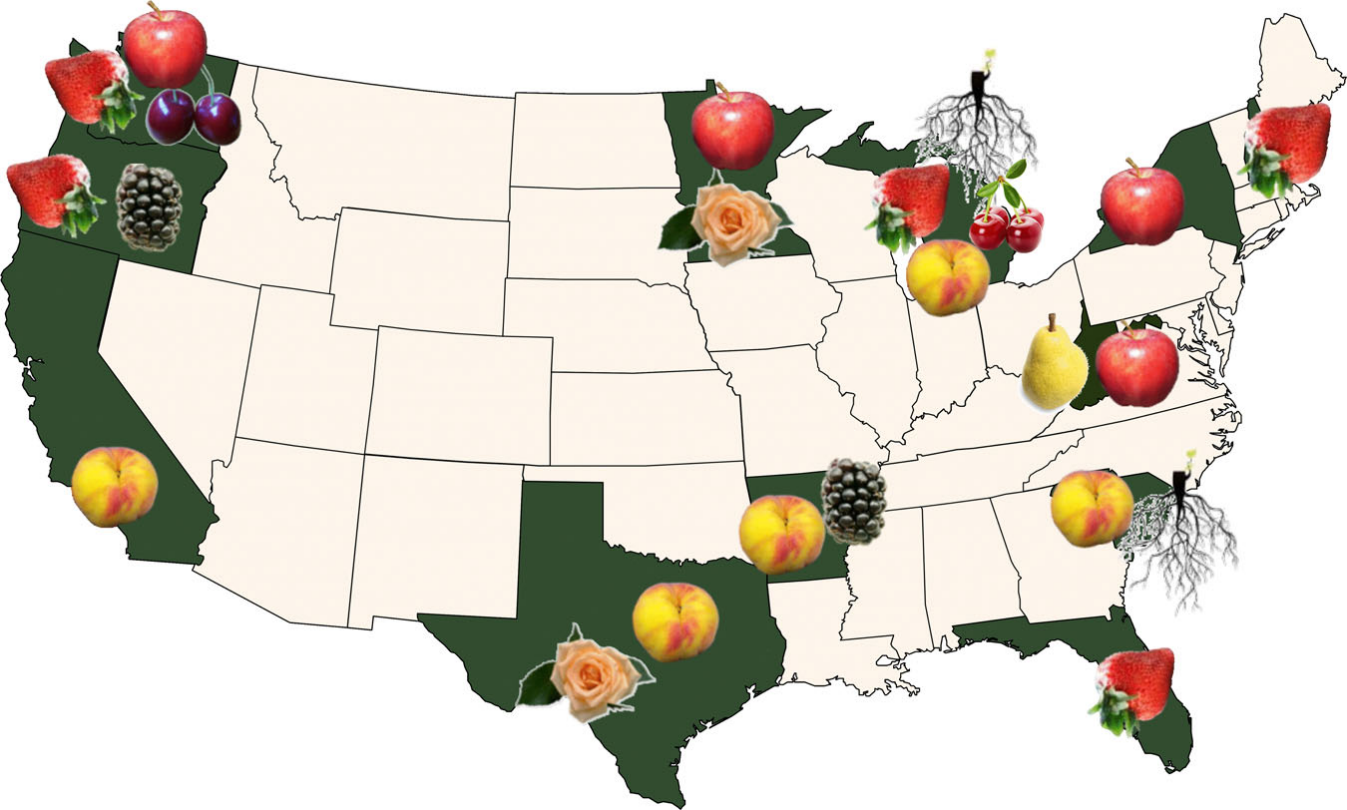
U.S.-wide demonstration breeding programs. *apple* Univ. of Minn., Wash. State Univ., Cornell Univ., USDA-ARS Kearneysville, WV; *peach* Clemson Univ., Univ. of Ark., Texas A&M Univ., Univ. Calif. – Davis; *sweet cherry* Wash. State Univ.; *sour cherry* Mich. State Univ.; *strawberry* USDA-ARS Corvallis, OR, Mich. State Univ., Univ. Florida, Univ. New Hampshire; *blackberry* Univ. of Ark., USDA-ARS Corvallis, OR; *pear* USDA-ARS Kearneysville, WV; *rose* Univ. of Minn., Texas A&M Univ.; *Prunus rootstock* Clemson Univ., Mich. State Univ. The two “newly adopting” breeding programs that participated in cost analyses were strawberry at Wash. State Univ. and peach at Mich. State Univ.

The RosBREED approach envisioned that the common breeding needs of diverse rosaceous crops for genomics information and diagnostic tools could be effectively addressed by energetic, large-scale, community-wide research and extension efforts. Permeating both RosBREED projects was an emphasis on collaboration and coordination of participants nationally and internationally (including participants in FruitBreedomics) with a committed focus on breeding outcomes (Supplementary Tables S1 and S2). Twelve breeding programs in RosBREED 1 and 22 breeding programs in RosBREED 2 served as “demonstration breeding programs” (Fig. 1). These programs identified their crop-specific needs and opportunities, highlighted appropriate germplasm, conducted phenotyping, searched for valuable trait loci, applied DNA-based information to breeding decisions, hosted breeding trainees, and served as examples for other rosaceous crop breeding programs.

The RosBREED approach featured a multi-stage pipeline to bridge the chasm between genomics resources and knowledge and practical application of DNA information in breeding programs^14^. Some stages were science-driven while others were logistic or socio-economic. Target traits were identified based on stakeholder input and socio-economic analyses. Next, genomic regions associated with such traits and their genetic linkage relationships with other trait-influencing genomic regions were investigated and validated. High-impact, large-effect trait loci were converted into trait-predictive DNA tests. The next stages addressed bottlenecks that hindered efficient, accessible, and affordable routine use of MAS. DNA tests were trialed and then used routinely to detect desirable alleles in breeding program germplasm. The term “jewels in the genome” was coined to communicate these deliverables to lay audiences via the analogy that “jewels” represented valuable alleles discovered and characterized in the crop’s genome. The jewels were then “polished” by being made detectable in each individual’s genome (i.e., by developing new DNA tests), put into routine breeding application via DNA test application, and then this new knowledge was incorporated into breeding decisions^16^. Expansions of the pipeline in RosBREED 2 included investigating methods to combine valuable alleles from multiple sources into single individuals over multiple generations, to explore the stability of QTLs across environments, and to test the utility of integrating genome-wide prediction methods, especially for traits not governed by large-effect loci^17^. In addition to these applications of DNA-based information regarding performance-predictive evaluation, RosBREED also improved methods for characterizing breeding germplasm for identity and relatedness.

The two RosBREED projects sought efficiencies for all stages of breeding germplasm, from pre-breeding generations for germplasm enhancement, to breeding parents, seedling families, elite selections, and released cultivars. Targets therefore included: (1) introgression, pyramiding, and combining of valuable alleles, especially from wild crop sources; (2) parent pool selection, cross design, and seedling selection based on large-effect trait loci (via locus-specific DNA tests) and many small-effect loci (via genome-wide selection); (3) G×E performance predictions to inform elite selection advancement and cultivar deployment across multiple environments; and (4) DNA fingerprinting assays for identity verification and more accurate mapping of relatedness at all germplasm levels. RosBREED researchers and collaborators also partnered with the GDR, to enhance access to publicly available genomics, genetics, and breeding data and data-mining tools to facilitate basic, translational, and applied research in the Rosaceae. The GDR provided the research community with data and resources for data mining, and as results were generated, RosBREED data were integrated, archived, and curated with other data.

## Genomics and socio-economics knowledge informs all stages of cultivar development

RosBREED enhanced development of powerful database resources, new knowledge, and useful tools to provide the foundational building blocks to help implement DNA-informed breeding in U.S rosaceous crop breeding programs. The RosBREED “pipeline” showcases the steps taken to implement its routine application for high priority attributes. Examples of DNA-informed breeding use by project participants are presented for all breeding stages, including pre-breeding for disease resistance, parental and seedling selection, and elite selection advancement.

### Trait level prioritization

It is crucial for breeders to focus efforts and resources on crop attributes that have high value throughout the supply chain. This focus is especially important when considering the initial investment needed to implement DNA-informed breeding, which requires extensive technical knowledge, trained personnel, and sufficient financial resources. Socio-economics research in RosBREED identified these high-impact targets by systematically quantifying the relative value of achieving incremental improvements in a range of traits using a willingness-to-pay approach with three sectors of the supply chain: producers, marketing intermediaries, and consumers.

Willingness to pay was elicited from supply chain members for numerous quality traits of four rosaceous fresh market fruit crops^19–21^, with crop-specific investigations of apple^22,23^, peach^24,25^, sweet and sour cherry^26^, and strawberry^27^. Across crops, results indicated consumers were willing to pay a price premium for flavor and textural components over fruit appearance attributes like size and external/internal color. Across crops, market intermediaries were willing to pay the highest price premiums for fruit quality traits associated with handling, like firmness and shelf life, or with U.S. grades and standards like size and external appearance. For producers, price premiums were highest for improvement in traits like flavor and texture, but the relative value producers placed on specific traits varied depending on crop. Highest premium traits identified by producers for apple were improved shelf life, flavor, and crispness; for peach, improved flavor, external color, and external appearance; for sweet cherry, improved size, flavor, and firmness; for strawberry, improved flavor, firmness, and external color. Interestingly, the traits prioritized by producers represented a combination of those identified by consumers and market intermediaries. In sum, systematic investigations reinforced the experience of rosaceous crop breeding programs—comprehensively and simultaneously addressing the priorities of all their supply chain stakeholders is a massive and dynamic challenge^28,29^. The challenge is exacerbated by the high resource and time costs of phenotyping to reliably evaluate traits with complex genetics and significant environmental interactions. The situation was rendered even more complex when industry stakeholders developing RosBREED 2 identified a key project goal of combining disease resistance and horticultural quality in new rosaceous cultivars^17,30^. However, the socio-economics information gained on trait priorities provided strong support to direct RosBREED resources to those genomic discoveries of highest relative value to stakeholders.

### Strategies for discovery of genetics knowledge

RosBREED-generated new genetics knowledge for high-priority traits. Enabling technologies had to be adopted or developed to provide genome-wide genetic information relevant to breeding germplasm. The project’s centralized support for these enabling technologies, such as pedigree-based analysis (PBA), standardized phenotyping, and single nucleotide polymorphism (SNP) arrays, united researchers and breeding programs within and across crops. These effective new approaches provided a common operating system for Rosaceae genetics advances and its subsequent translation into crop genetic improvement. For the polyploid crops with complicated inheritance patterns, genetic resources developed by other research teams were leveraged to enable advances.

#### Reference germplasm sets for U.S. breeding

**Fig. 2:**
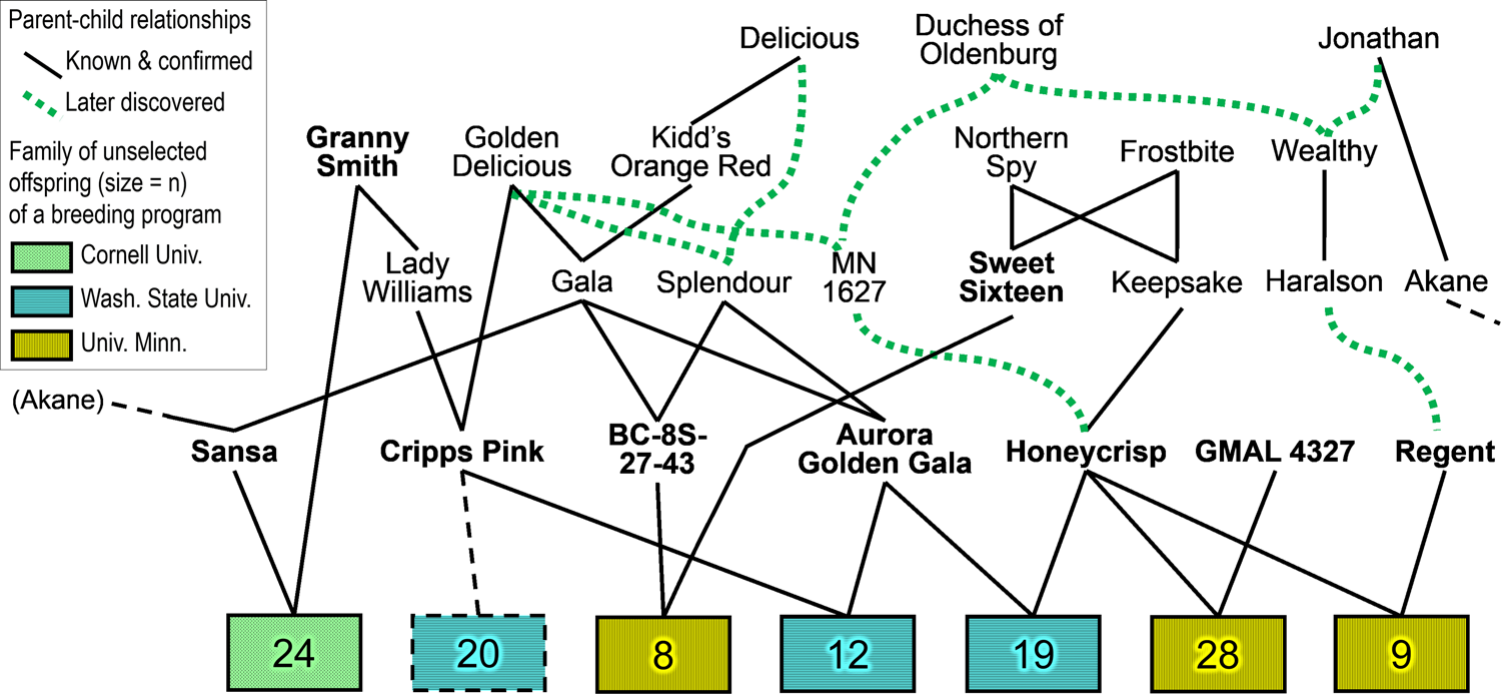
RosBREED’s reference germplasm sets were designed to represent alleles of important breeding parents (IBPs) to support simultaneous QTL discovery and validation in breeding program germplasm. A subset of the apple Crop Reference Set is shown here, which involved collaboration among breeding programs from three institutions. IBPs in this subset are in bold. “Honeycrisp” was represented directly by immediate descendant F_1_ families (here shown with 19 + 28 + 9 offspring) as well as indirectly by closely related families (such as the “Sweet Sixteen” × BC-8S-27-43 family). “Cripps Pink” and “Granny Smith” were each represented by the other’s direct F_1_ families. The dashed lines and family indicate further representation of “Cripps Pink” with the possible inclusion of 20 offspring in a Breeding Pedigree Set family. IBPs were also often represented by other families via ancestral relationships (e.g., “Sansa” was represented by all families also descended from “Golden Delicious” or “Delicious”). The germplasm sets were chosen based on pedigree knowledge at the time. Much additional allelic representation was provided by many other pedigree connections later discovered, such as “Golden Delicious” being a grandparent of “Honeycrisp” and “Splendour” being the offspring of “Golden Delicious” and “Delicious”. Some pedigree records were also corrected (e.g., “Regent” was discovered to not be “Duchess of Oldenburg” × “Delicious” but rather the child of “Haralson” and “McIntosh”)

For each crop, a crop reference set was collaboratively chosen by demonstration breeding programs. Each set consisted of germplasm representing important breeding parents and their immediate offspring, with many known pedigree connections among parents, to provide replication of parental alleles^31^ (Fig. 2). As each breeding program had its own distinctive germplasm, demonstration breeders also added and analyzed their own unique plant materials (called breeding pedigree sets) that were pedigree-connected to the crop reference sets. In total, the reference germplasm sets comprised almost 1000 individuals each for apple, peach^31^, and strawberry^32^, and almost 500 each for sweet cherry and sour cherry^31^.

#### Standardized phenotyping

Phenotypic evaluation of the reference germplasm sets was conducted in a standardized manner for each crop across the multiple breeding programs and multiple years. This consistent approach enabled combining of data sets across programs for improved statistical power to ensure that trait–DNA associations could be accurately determined. Each RosBREED 1 crop breeding team of apple, peach, strawberry, sweet cherry, and sour cherry agreed on the traits to be evaluated and then developed and documented standardized protocols to measure these plant and fruit characteristics. They compiled extensive phenotypic data sets for fruit quality and other critical traits over three years and multiple locations. Standardized phenotyping was conducted for 37 traits for strawberry^32^, 43 for apple^33,34^, 29 for peach^35^, 19 for sweet cherry^36^, and 35 for sour cherry^37^. Standardized phenotyping was added for blackberry and expanded for peach in RosBREED 2. For blackberry, standardized phenotyping protocols were developed and followed for 17 traits^38^. For peach, a post-harvest phenotyping protocol, previously developed by Crisosto et al.^39^, was applied to 480 individuals from three public peach breeding programs in Texas, South Carolina, and Arkansas, collecting 27 measures for eight key traits. Detailed phenotyping protocols are available at www.rosbreed.org and publicly available RosBREED-generated phenotypic data can be accessed in the GDR using the “Search Trait Evaluation” page to query qualitative or quantitative traits.

Disease resistance was also evaluated in RosBREED 2 and standard protocols were developed as illustrated here for three tree fruit diseases. For peach, skin and flesh response to brown rot (caused by *Monolinia* spp.) was evaluated on 164 individuals^40,41^ and a modified detached-leaf bioassay^42^ was used to record leaf resistance to bacterial spot (caused by *Xanthomonas arboricola* pv. *pruni*) on 360 individuals from the South Carolina and Arkansas programs^43^. For cherry leaf spot disease of sour cherry (caused by *Blumeriella jaapii*), a phenotyping scale was developed and used to rate the disease severity and progression of 206 individuals, many of which were in pedigree-connected families^44^. For apple fire blight (caused by *Erwinia amylovora*), a phenotyping scale was developed based on incidence and disease severity and used to classify 94 apple cultivars into three resistance/susceptibility groups^45^. In all cases, this phenotypic data was used to determine the inheritance of the disease reaction.

#### Standardized genome-wide genotypic data via Rosaceae SNP arrays

An illustrative example of international collaboration was the development of SNP-based genome-scanning capability for each crop in RosBREED 1. These arrays filled a critical technology gap, because at the time there were no efficient genome-wide genotyping platforms available for any rosaceous crop and genotyping was laboriously done using a limited number of simple sequence repeat (SSR) markers. Design of these three arrays was possible due to the newly available reference genome assemblies developed for apple^5^, peach^10^, and diploid strawberry^11,46^ by the three international crop communities working in parallel to RosBREED. Plans for the genome scans were devised and agreed upon at an International SNP Summit in 2010, followed by SNP crop-specific arrays designed through collaborative efforts led by researchers in the U.S. (strawberry^47^, cherry^48^), New Zealand (apple^49^), and Italy (peach^50^). These SNP arrays, commercialized by U.S.-based vendors and used worldwide, provided a robust international resource for genome-wide genotyping, greatly facilitating comparison of results among research teams.

The 6K cherry^48^, 8K apple^49^, and 9K peach^50^ arrays were all implemented on the Illumina Infinium® platform. Array design began with SNP discovery in relevant germplasm panels, using high-throughput DNA sequence sets aligned to the respective reference genomes, where the peach genome sequence was used as a proxy for cherry. For the 9K peach SNP array, SNPs distributed across all eight peach chromosomes with an average spacing of 26.7 kb between SNPs were chosen^50^. A peach consensus map, based on 9K SNP array markers shared across five genetic linkage maps, was subsequently constructed to provide valuable information on marker order and genetic position and to aid in estimation of genetic positions of unmapped markers^51^. A similar strategy of evenly spaced SNPs was used for the sweet and sour cherry array, for which the peach genome served as a proxy because a cherry sequence was unavailable at the time^48^. As sour cherry has both a sweet cherry-derived and a *P. fruticosa*-derived sub-genome, the final SNP choice involved an extra step to assure sampling of the *P. fruticosa* sub-genome. Linkage maps constructed using two sweet cherry populations assigned genetic positions for the SNPs, confirming the high level of synteny between peach and cherry^52^. The apple SNP array targeted focal points evenly spaced at intervals across the apple linkage map (~every 1 cM)^49^. At each focal point, 4–10 clustered SNPs were chosen to enable the construction of haplotypes for each focal point and therefore the potential identification of multiple haplotypes at each genome location.

In strawberry, due to the complexities of octoploidy and the expectation of an elevated marker failure rate, the Affymetrix® Axiom® platform was chosen as it could accommodate in excess of 90K markers^47^. SNPs were chosen for the 90K array where one of the two marker alleles was predicted to be present in and segregate from only one sub-genome (the marker sub-genome) as the marker allele was predicted to be either homozygous or absent in the three other subgenomes. The 90K Axiom® strawberry SNP array was the first genotyping array commercially available for an octoploid organism^47^ and the linkage positions for many of the SNPs were subsequently determined using various *Fragaria* species and populations^53–55^. The publicly available genotype data from RosBREED for apple, peach, cherry, and strawberry can be accessed on GDR via the SNP genotype search page. In addition, the SNP array data sets can be downloaded in the SNP array section of the genus/species pages and are displayed as tracks in JBrowse^56^. The SNPs are routinely aligned to the new genome assemblies when they become available.

The initial arrays developed were extensively used by RosBREED participants and the worldwide research community for trait locus identification and validation. Their utility, along with continued advances in genomics information and technologies, led to various groups designing and commercializing the next generation arrays with increased numbers of markers for all four crops: a 20K Infinium® and a 489K Axiom® array for apple^57,58^, a + 9K SNP “add on” to the 9K peach array^59^, a + 9K “add on” to the 6K cherry array^60^, and a 50K strawberry array based on the octoploid sequence assembly^61^. Axiom® arrays for pear (70K array^62^ and 200K array^63^) and rose (68K array^64^) developed by other research groups enabled genetic discovery and validation of disease resistance loci.

Alternative genome-scanning approaches for genotyping a lower density of genome-wide SNPs at a reduced sample price were adopted from the previously designed platforms to support breeder’s use of genome-wide selection for seedling selection. For strawberry, a more cost-efficient 35K array that contained SNPs from the 90K Axiom® SNP array informative across multiple breeding programs was developed for use in genome-wide selection by the Univ. of Florida strawberry demonstration breeding program^65^. For peach, a SeqSNP (LGC Genomics) targeted genotyping-by-sequencing strategy with a lower density and tailored set of SNPs was developed. This peach SeqSNP assay consisted of 3,000 informative SNPs from the 9 + 9K peach SNP array, including previously identified SNPs associated with traits of interest (K. Gasic, pers. comm.). As in strawberry, the SeqSNP assay is now in use for peach genome-wide selection.

#### Pedigree-based analysis (PBA) for trait locus discovery, characterization, and validation

A unique and powerful aspect of the RosBREED approach was the use and advancement of statistical software for QTL discovery, characterization, and validation as well as estimation of QTL performance and breeding values. FlexQTL™ software (www.flexqtl.nl)^66^, developed by researchers at Wageningen University and Research, the Netherlands, based on an idea of Uimari & Sillanpää^67^, follows a Bayesian approach and can simultaneously analyze data from various-sized pedigreed populations comprising multiple generations connected by common ancestry—a situation typical of heterozygous rosaceous breeding populations. Simultaneous QTL discovery and validation in multiple genetic backgrounds in available fruiting populations was thereby enabled, leading to discovery and characterization of functional alleles in the breeding-relevant germplasm that for RosBREED was represented by the crop reference sets and breeding pedigree sets. Published MTLs and QTLs, including those from RosBREED, are integrated into a search page on the GDR that links them to many other GDR resources including trait descriptions, screening methods, map positions, and associated markers.

The core of a pedigree-based analysis is provided by FlexQTL^TM^, which is used for the discovery and characterization of QTLs, curation and characterization of input data, phasing of markers, and estimation of Identity-by-Descent probabilities^66,68–70^. A suite of additional software has been developed to further facilitate, deepen, and widen the genetic analyses and interpretations. To prepare the high-resolution genotypic input data, several tools are utilized^71^. “ASSIsT”^72^ supports the calling and filtering of SNP markers from the Illumina Infinium platform and “FlexQTLDataPrepper” facilitates creation of the actual marker data input file^71^. Next, software was designed to ease interpretation of large SNP marker data sets and reduce computation time by aggregating genetic information from large sets of bi-allelic SNP markers into a condensed data set of multi-allelic haploblock markers. “VisualFlexQTL” facilitates sizing of haploblocks^71^, while “PediHaplotyper”^73^ assigns their haplotypes. Visualization of pedigree data is facilitated by “Pedimap”^74^. While this suite of software was developed primarily for QTL analyses, FlexQTL^TM^’s phasing functionality on breeding germplasm and PediHaplotyper’s ability to condense marker information supported studies on unraveling additional genetic relationships among cultivars. FlexQTL^TM^ and all other published PBA software is publicly available. To visualize genome-wide genetic information of ancestry, relatedness, and trait locus alleles^75^, software is under development (L. Bianco & C. Peace, pers. comm.). A workflow for extensive SNP data curation (including verifying genotype calls, inheritance, and marker order on genetic maps) was developed^71^. Publicly available genotypic and haplotypic data from RosBREED was archived^71^ and can also be accessed in the GDR via the haplotype block search page where the SNP markers within the haploblocks and their genomic and genetic positions are hyperlinked to JBrowse^56^ and MapViewer^76^.

#### Strategies for polyploid crops

Alternative analytical software was required for the polyploid crops of blackberry, rose, and sour cherry. As strawberry is an allo-octoploid, disomic inheritance of the sub-genome specific markers enabled strawberry researchers to use software for diploids such as FlexQTL™ and accompanying software for QTL analysis. In contrast, sour cherry and tetraploid rose can exhibit both disomic and tetrasomic inheritance, while blackberry only exhibits polysomic inheritance. Therefore, different analytical software was used to account for their higher allelic dosages and random meiotic pairing^77,78^. New software applications were used to assign dosage to offspring^79^, including the R packages “fitTetra” and “fitpoly”, with the latter capable of assigning dosage to any ploidy level and “fitTetra” suitable for tetraploids only^77,80,81^. Unlike other programs, both “fitTetra” and “fitpoly” were designed to account for abnormal segregation ratios and therefore were effective for rose and sour cherry. The R package “polymapR”^77,79^ was used for rose to create maps for tetraploid × tetraploid families with random meiotic pairing, and accommodated preferential chromosome pairing during meiosis^82,83^.

### Jewels in the genome

The most sought-after pieces of genetic information by RosBREED were loci with large effects on high-priority traits that segregated in breeding germplasm. Such “jewels in the genome” were often those discovered by project researchers via PBA in U.S. breeding germplasm that had been genome-scanned with SNP arrays and undergone standardized phenotyping. MTLs and QTLs reported by Rosaceae scientists around the world and validated in U.S. breeding germplasm were also valuable targets when their alleles were expected or shown to segregate in local families. The DNA information for the alleles of these trait loci was then used to develop DNA tests^84^ or refine existing DNA tests for diagnostic use on U.S. breeding germplasm (Table 2).

**Table 2:** Loci with desirable alleles discovered and/or validated and DNA tests developed through RosBREED efforts, including international collaborations.

Crop	Trait	Loci with desirable alleles discovered and/or validated; DNA test developed
Fruit quality		
Apple	Fruit texture (ethylene)	Validated, *MdACS1* (LG15)^85,86^; DNA test^89^
	Fruit texture (ethylene)	Validated, *MdACO1* (LG10)^85,86^; DNA test^89^
	Fruit texture (firmness)	Validated, *MdPG1* (LG10)^87^; DNA test^89^
	Fruit texture (firmness)	Validated, *MdExp7* (LG1)^196^; DNA test^196^
	Fruit texture (crispness)	Validated, *Ma*^197^; DNA test^89^
	Fruit acidity	Validated, *Ma* + *Ma3* (LG16 + LG8)^94^; DNA test^89^
	Fruit sweetness	Validated, *Md-LG1Fru* (LG1)^198^; DNA test^89^
	Bitter pit susceptibility	Validated, *Bp1* (LG16)^199^; DNA test^89^
	Fruit skin blush	Validated, *Rf* (LG9)^200^; DNA test^89^
	Fruit flesh color	Validated, *MYB110* (LG17)^88,201,202^; DNA test^88^
	Soft scald	Discovered, “Honeycrisp” source (LG2 & 12)^95^
Peach	Fruit skin blush	Validated, *R*_*f*_ (LG3)^203^; DNA test^203^
	Fruit acidity/flavor	Validated, *D* (LG5)^103,204^ and flavor (LG7)^205^
	Peach vs. nectarine	Validated, *G* (LG5)^102^; DNA test^102^
	Yellow vs. white flesh	Validated, *Y* (LG1)^101^
	Fruit texture types	Validated, *F-M* and “*SMF*” (LG4)^100,206,207^; DNA test (K. Gasic, pers. comm.)
	Fruit sweetness	Validated, (LG4)^104,208^
	Fruit size	Validated, (LG4)^105^, (LG6)^106^
Strawberry	Fruity aroma	Validated, *FaFAD1* (LG3B)^117^; DNA test^125^
	Fruit sherry aroma	Validated, *FaOMT*^119^; DNA test^119^
	Fruit sweetness	Discovered, QTL^116^
	Fruit pH	Discovered, QTL^116^
	Fruit acidity	Discovered, QTL^116^
Sweet cherry	Fruit size	Validated, *FW_G2* (LG2)^166^; DNA test^136^
	Fruit color type	Validated, *R*_*f*_ (LG3)^134^; DNA test^167^
	Fruit firmness	Discovered, *qP-FF4.1* (LG4)^138^
	Fruit sweetness	Discovered, (LG2) (C. Peace, pers. comm.)
Sour cherry	Fruit flesh color	Validated, *MYB10* (LG3)^135^; DNA test^135^
Blackberry	Fruit sweetness	Discovered, *qSSC-Ruh-ch1.1*^152^; DNA test^152^
Disease resistances and physiological disorders		
Apple	Blue mold	Discovered, *M. sieversii* PI source (LG3)^98^; DNA test^157^
	Powdery mildew—foliar	Validated, “White Angel” source (LG8)^90^; DNA test^209^
	Scab	Validated, *M. floribunda* source (LG1)^91^; DNA tests^89^
	Scab	Discovered, “Honeycrisp” & “Wildung” sources (LG1, LG15)^210^
	Scab	Validated, R12704-7A source, *Rvi2* (=*Vh2*)^211^; *Rvi4* (=*Vh4*)^211^; DNA tests^89,92^
	Scab	Discovered*, M. sieversii* source, *Rvi8* (=*Vh8*)^93^; DNA test^93^
	Fire blight	Validated, “Cox’s Orange Pippin” source (LG7)^212^
	Fire blight	Validated, “Splendour” (LG5) source^99^
	Fire blight	Discovered and/or validated, various sources (LG6, 7, 15) (S. Kostick, pers. comm.)
	Zonal leaf chlorosis	Discovered, “Honeycrisp” source (LG9)^97^
Peach	Bacterial spot—fruit	Discovered, “Clayton” source (LG1 & 6)^112,213^; validated, DNA test^164^
Strawberry	Fruit & crown rot	Validated*, FaRCa1* (LB6B)^121^; DNA test^214^
	Crown rot	Discovered, *FaRCg1* (LG6B)^122^; DNA test^122^
	Root & crown rot	Validated, *FaRPc2* (LG7D)^123^; DNA test^126^
	Red stele	Validated *Rpf1*^215^; DNA test^216^
	Angular leaf spot	Validated*, FaRXf1* (LG6D)^124^; DNA tests^124,127^
Sweet cherry	Powdery mildew—fruit & foliar	Discovered (fruit), validated (leaf), *Pmr1* (LG5) (C. Peace, pers. comm.); DNA test (C. Peace, pers. comm.)
Sour cherry	Cherry leaf spot	Discovered, (LG4)^139^; DNA test^139^
Pear	Fire blight	Validated, “Moonglow” (LG2) source^147^
	Fire blight	Discovered, “Old Home” source (LG2)^147^
	Fire blight	Discovered, (LG2)^147^
Rose	Black spot	Discovered, *Rdr4* (LG5-1)^82^
	Black spot	Mapped, *Rdr3* (LG6-2)^83^; DNA test^83^
	Black spot	Discovered, partial resistance (LG3)^145^
Phenology and productivity		
Apple	Cross-compatibility	DNA test, *S* (LG17)^217^
Peach	Bloom timing	Validated, (LG1, LG4, LG7)^104,218,219^
	Maturity timing	Validated, *G4Mat* (LG4)^104,108^; DNA test developed (K. Gasic, pers. comm.)
Strawberry	Perpetual flowering	Validated, *FaPFRU* (LG4A)^115^; sub-genome localization^116^; DNA test validation^128^
Sweet cherry	Self-compatibility	DNA test, *S* (LG6, S4, allele)^132^
	Cross-compatibility	DNA test, *S* (LG6)^130^
	Bloom timing	Discovered, (LG1)^140^
Sour cherry	Bloom timing	Validated, (LG4)^78^

Traits types are fruit quality, disease resistances, and physiological disorders, phenology, and productivity.

#### Apple jewels in the genome

RosBREED researchers took advantage of considerable prior progress in the identification of large-effect trait loci associated with valuable traits in apple. Such traits with available DNA tests included fruit firmness, texture, and crispness (*MdACS1*^85,86^, *MdACO1*^85,86^, *MdPG1*^87^), fruit skin and flesh color [*Rf* (C. Peace, pers. comm.); *MYB110*^88^], acidity (*Ma*^89^), “White Angel”-derived powdery mildew tolerance^90^, *M. floribunda*-derived scab resistance (*Rvi6* = *Vf*)^91^, and the previously characterized scab resistance loci of *Vh2*^92^, *Vh4*^92^, and *Vh8*^93^. These loci were validated within the apple crop reference and breeding pedigree sets. Verma et al.^94^ further elucidated the genetic control of fruit acidity in the germplasm of a U.S. breeding program by characterizing functional alleles for two QTLs, *Ma* and *Ma3*, and developing an additive allele dosage model for these two loci.

Due to the importance of “Honeycrisp” as a parent in all demonstration apple breeding programs, identifying and characterizing loci for important traits heterozygous in this cultivar was a high priority. Newly identified alleles inherited from “Honeycrisp” influencing soft scald and soggy breakdown^95^, scab tolerance^96^, and zonal leaf chlorosis^97^ were identified. In RosBREED 2, additional loci for resistance to two important diseases were identified. A large-effect QTL for resistance to post-harvest blue mold infection of fruit, caused by *Penicillium expansum*, was discovered in a family descended from a *M. sieversii*^98^ accession, and a fire blight resistance QTL was discovered segregating from “Splendour”^99^. A compilation of SNPs associated with apple MTLs and QTLs was made and individual SNP assays were developed and tested^89^.

#### Peach jewels in the genome

In peach, several fruit appearance and eating quality traits controlled by MTLs had DNA tests developed outside of RosBREED, especially by researchers in FruitBreedomics. Such MTLs included those for freestone vs. clingstone and fruit flesh softening (*F-M*^100^ and *“SMF*” loci), yellow vs. white fruit flesh color (*Y* locus)^101^, nectarine (glabrous fruit skin) vs. peach (pubescent skin) fruit type (*G* locus)^102^, and fruit acidity (*D* locus)^103^ (Table 2). QTLs detected for bloom and maturity timing and fruit size^104–107^ were validated. Haplotype analyses in the QTL regions for sweetness and skin blush identified diagnostic SNP haplotypes and specific SNPs associated with desirable QTL alleles^108^. In addition, GWAS analysis revealed stable SNP associations over two evaluation years for four loci: *F-M*, *G*, *D*, and redness around the pit (*Cs*) (C. da Silva Linge, pers. comm.).

In RosBREED 2, with 426 additional peach individuals genome-scanned with the newly developed peach 9 + 9K SNP peach array, there were sufficient markers to perform GWAS using GModel and FarmCPU (Fixed and random model Circulating Probability Unification)^109,110^. An SNP marker associated with the post-harvest traits of mealiness, browning, bleeding, expressible juice, and texture was detected (linked to a QTL cluster associated also with bloom and maturity timing, stone-flesh adhesion, and fruit softening), while an SNP on another chromosome was associated with all traits except bleeding^111^. These two loci became targets for DNA test development.

For fruit disease resistance, two loci were identified in offspring of “Clayton” associated with fruit tolerance to bacterial spot, caused by *Xanthomonas arboricola* pv. *pruni*, one of the most important peach diseases in humid production regions^112^. SNPs associated with bacterial spot foliar resistance were identified on all chromosomes using various approaches (biparental families^112^ and GWAS^113^; K. Gasic, pers. comm.). For fruit brown rot tolerance, 26 cultivars and families derived from nine crosses with “Bolinha” as the resistance source were evaluated in two seasons for disease severity index in wounded and non-wounded fruit. SNP markers significantly associated with relatively small components of the total phenotypic variance of brown rot response were identified; genome-wide selection might be a more effective breeding strategy for improving this trait^114^.

#### Strawberry jewels in the genome

A large-effect QTL for perpetual flowering, previously reported^115^, was validated in RosBREED germplasm^116^, along with two other previously identified loci associated with fruity aroma^117,118^ and sherry aroma^119^. Due to the short seed-to-seed cycle in strawberry, mapping populations could be rapidly created upon the discovery of interesting phenotypes. For example, resistance evaluation for charcoal rot, caused by *Macrophomina phaseolina*, led to the creation of pedigree-connected populations and the discovery of new MTLs for resistance within a two-year period^120^. Disease resistance QTLs discovered and validated included *Ca1* for fruit and crown rot caused by *Colletotrichum acutatum*^121^, *Cg1* for crown rot caused by *Colletotrichum gloeosporioides*^122^, *Pc2* for root and crown rot caused by *Phytophthora cactorum*^123^, and *Xf1* for bacterial angular leaf spot caused by *Xanthomonas fragariae*^124^. QTLs were also identified for horticultural quality traits such as fruit sweetness and acidity^116^.

In addition to the identification of QTLs, new DNA tests were developed and existing DNA tests were validated for strawberry in diverse collections of germplasm^124–128^. As a resource for strawberry breeding programs interested in conducting MAS, details on strawberry DNA tests developed by RosBREED and other strawberry research programs were distilled into a compendium^129^. This Strawberry DNA Test Handbook exists as a downloadable document on the GDR and is updated as new or improved tests are reported^12,129^.

#### Cherry jewels in the genome

The use of DNA markers to determine self-incompatibility genotypes in sweet and sour cherry was one of the first widely adopted uses of DNA information in rosaceous crops. In addition to genotyping for *S* alleles in general^130,131^, breeders specifically sought to develop self-compatible cultivars. DNA diagnostic tests were available for detecting the “Stella”-derived S_4_’ allele for self-compatibility in sweet cherry^132^, the “Cristobalina”-derived self-compatibility modifier allele^133^ in sweet cherry, and the series of self- compatibility alleles in sour cherry^131^.

As with apple and peach, several large-effect loci previously reported for important sweet cherry traits were fast-tracked for DNA test development. These MTLs and QTLs included *MYB10*, associated with fruit color in sweet^134^ and sour cherry^135^ and orthologous to the major anthocyanin *MYB10* locus of apple and peach. A QTL was reported for fruit size^136,137^ and another QTL was detected for fruit firmness^138^; the “small” and “soft” alleles, respectively, were derived from wild germplasm. In RosBREED 2, an MTL reported previously for controlling foliar powdery mildew resistance was determined to also condition fruit resistance to the disease, and several supposedly diverse resistance sources were discovered to carry the same allele (C. Peace, pers. comm.). In sour cherry, an MTL for cherry leaf spot resistance was identified, with the desirable allele derived from *P. canescens*^139^.

QTLs were identified for bloom and maturity timing for sweet and sour cherry in the same genomic region as for peach; in sour cherry, the late-flowering alleles appear to be from the *P. fruticosa* sub-genome^78^. The very early bloom timing in the sweet cherry landrace “Cristobalina” associated with a very low chilling requirement was shown to be due to homozygosity for a large-effect QTL on a different chromosome that co-located with dormancy genes previously identified in peach^140^.

#### Rose jewels in the genome

A singular goal was pursued for rose in RosBREED 2: Enable DNA-informed breeding for black spot, caused by *Diplocarpon rosae*, one of the most devastating foliar diseases of outdoor-grown roses^141^. This task was especially challenging due to multiple physiologic races of the fungus^142,143^. By 2014, three resistance loci had been identified (*Rdr1, Rdr2*, and *Rdr3*)^142,143^ and a DNA test was available for *Rdr3* that mapped approximately 9.3 cM away from the locus^144^. As such, there was a fair chance with each generation for the diagnostic marker allele to lose coupling phase linkage with the *Rdr3* resistance allele, reducing confidence in the DNA test.

Further efforts focused on important resistance sources used by each rose demonstration breeding program. The Univ. of Minnesota breeding team sought to map *Rdr3* in the tetraploid rose “George Vancouver” and identify qualitative resistance from the tetraploid rose cultivar Brite Eyes^TM^. Using a “George Vancouver” biparental population, *Rdr3* was mapped and three new DNA tests were created that co-segregated completely with *Rdr3* and were easier to interpret than the previous test^83^. The broad resistance from Brite Eyes^TM^ (resistance to all but one of the 13 *D. rosae* races) made it an attractive source for breeding^82^, and mapping revealed a novel locus, *Rdr4*^82^. The Texas A&M Univ. breeding team identified and characterized a QTL conferring partial resistance in diploid roses based on a PBA study with related families derived from resistant breeding selections^145^.

#### Pear jewels in the genome

The RosBREED goal for pear was to identify alleles conferring resistance to fire blight, caused by the bacterial pathogen *Erwinia amylovora*, in USDA-ARS pear scion breeding program germplasm^146^. Using three biparental breeding populations, a QTL was identified in a similar genomic location for each family^147^. This trait locus had been reported in previous studies^148–150^ and its validation in RosBREED germplasm confirmed its utility as the most informative locus with the most effective resistance allele(s) available. Candidate resistance genes were identified using the recently available chromosome-scale pear genome assembled using a map generated from one of the RosBREED 2 mapping families^151^.

#### Blackberry jewels in the genome

Despite the resource and technological lag behind other rosaceous crops, RosBREED contributed to blackberry genetics by utilizing the HybSeq approach to identify alleles associated with fruit sweetness (soluble solids content) and develop DNA tests for this trait^152^. Breeder-friendly haplotype-specific KASP assays were developed and validated in four environments. A total of 48 markers were significantly associated with sweetness in at least one environment and 20 were stable in at least two environments. One sweetness-associated marker-haplotype was identified that accounted for an approximate 1.5° Brix increase in three environments. These sweetness markers identified by RosBREED 2 represent the first fruit quality marker-trait associations identified in blackberry.

#### Commercial service providers for DNA testing

Access to DNA-based diagnostics services was a key challenge to widespread breeder adoption of DNA-informed breeding. This challenge was identified in a review of the first set of reported successes in Rosaceae^15^ and via Rosaceae breeder community surveys conducted by an external evaluator of RosBREED 1. Therefore, in the last three years of RosBREED 2, genotyping transitioned to commercial service providers. As part of the transition process, RosBREED researchers resolved logistical issues related to growing and sampling plants, organizing and transporting samples to processing labs, and identifying reliable and cost-efficient vendors and genetic marker platforms. To integrate into the key milestones and ongoing breeding activities of each program, a generic scheme was developed to streamline and coordinate the timing of critical steps: tissue sampling (by the breeder or service provider), DNA extraction (service provider), genotyping (service provider), results interpretation (service provider and/or breeder), and selection action (breeder). It was clearly shown that each test must be technically reliable in the vendor’s lab and is best confirmed to the satisfaction of the breeder client by using a set of control samples weeks or months ahead of time. Occasionally, previously reliable DNA tests required recalibration, e.g., following the incorporation of new germplasm into a breeding program.

### Pre-breeding: introgressing, pyramiding, and combining valuable alleles

RosBREED 2 included a pre-breeding objective to provide tools and new germplasm to breeders for efficiently accessing disease resistance alleles in exotic germplasm. The major steps were to identify disease-resistant loci, design DNA tests for resistance alleles, and introduce these alleles into sets of “intermediate” materials, called “multiple resistance allele donors”. To access alleles for resistance from multiple sources, many crosses and generations would be needed: two sources requiring one generation, three to four sources requiring two generations, and so on. The strategy of creating a “multiple resistance allele donor” was to enable breeders to make just one cross to provide these multiple resistances to the next, new-cultivar-containing generation, thereby markedly increasing breeding efficiency. Creating the “multiple resistance allele donors” was possible due to use of DNA tests to track the inheritance of target alleles for selection in large numbers of plants in each generation. “Pyramiding” was done to incorporate multiple resistance alleles for the same disease into single individuals to enhance resistance durability and “combining” was done to incorporate multiple resistance alleles for multiple diseases into single individuals.

The short seed-to-seed cycle in strawberry and the availability of DNA tests enabled rapid identification, pyramiding, and combining of target alleles for horticultural quality and disease resistance. For example, the Univ. of Florida strawberry demonstration breeding program made crosses over three consecutive generations to combine resistances to *Xanthomonas*, *Phytophthora*, and *Colletotrichum* and with the use of DNA tests was able to identify offspring with the desired multiple resistance alleles (V. Whitaker, pers. comm.).

In apple, the USDA-ARS apple demonstration pre-breeding program created “multiple resistance allele donors” for scab and fire blight by utilizing a quick-to-flower rapid-cycling transgenic line to significantly reduce the generation time^153–155^. This transgenic line expresses the FRUITFUL homolog (*BpMADS4)* from silver birch (*Betula pendula*) that reduces the juvenile phase of apple from 2–5 years to just 3–7 months^153^. Because the *BpMADS4* transgene behaves genetically as a single dominant heterozygous allele, in subsequent crosses it segregates 1:1 such that half of the resulting population inherit the *BpMADS4* transgene for reduced juvenility, and the other half are non-transgenic with normal growth and juvenility. In each generation, DNA tests were used to select for the target disease resistance alleles, presence of the *BpMADS4* transgene, and segregating fruit quality alleles. The introgression strategy was expanded by utilizing SNP array data to determine the genome-wide numbers and proportions of DNA segments inherited from unimproved ancestors (typically the resistance allele sources in wild relatives) for each offspring carrying the desired alleles^156^. For introgression of blue mold resistance from a *M*. *sieversii* accession over two generations, DNA testing reduced a family of 129 offspring to 43, then 20K array SNP genome-scanning reduced the pool of candidate parents for the next generation to just three individuals that had low proportions and few segments of *M*. *sieversii* genome as well as favorable recombination close to the resistance locus^157^. After achieving the desired allele accumulation, non-transgenic individuals no longer carrying the *BpMADS4* transgene but with desired trait locus alleles were selected for use as multiple resistance allele donors. This effort resulted in new breeding parents that contained resistance alleles for scab (at the *Rvi6* and *RviHC* loci) and fire blight (*FB5* and *FB7*) from multiple sources. Pollen of these donors containing the rapid-cycling transgene will be deposited at the USDA-ARS Fort Collins, Colo. Multiple disease resistance donors without the rapid-cycling transgene, and therefore not considered transgenic, are pending approval for deregulation. Upon approval, these non-transgenic individuals will be added to the USDA-ARS *Malus* genetic resources collection in Geneva, N.Y., providing apple breeders with access to breeding parents they can use to incorporate multiple disease alleles into new cultivars in just one generation.

### Parental and seedling selection

RosBREED provided new DNA information and tools to assist breeders in their decisions regarding families expected to contain new cultivars—which parents to choose, what cross combinations to make, and which resulting seedlings to select. Implementation of marker-assisted parental selection (MAPS) and MASS (marker-assisted seedling selection) with locus-specific DNA tests for high-priority traits (Table 2) is described below for five demonstration programs. Incorporating such DNA-informed breeding technologies requires significant costs for supplies, equipment, and labor. To help identify which DNA tests, among the increasing number available, would be most effective to achieve breeding goals for MASS, strategies were explored in RosBREED via empirical investigation of published successes and modeling of genetic gain efficiency. As part of the socio-economics effort in RosBREED, a decision-support tool was developed to allow practitioners to estimate costs and benefits in their specific program. The tool was evaluated for MAPS and MASS using data from five project programs. In addition, the effectiveness of genome-wide selection (GWS) was evaluated in three programs (apple, peach, and strawberry).

#### Decision-support strategies for genetic gain considerations

A review of reported MASS successes in Rosaceae breeding identified that the first effective applications used DNA tests for high-impact traits and targeted trait loci explaining a high proportion of phenotypic variation (i.e., MTLs or at least very large-effect QTLs)^15^. Genetic gain modeling was subsequently used to quantify the relative effectiveness of MASS compared to traditional phenotypic selection in alternative scenarios, such as where DNA tests explain a lesser degrees of variation and for traits with lower heritability^158^. Results of both analytical derivation and stochastic simulation provided the same outcome: genetic gain tends to be higher for MASS where the proportion of genotypic variance explained by a DNA test is greater than the broad-sense heritability^158^.

#### Decision-support tools for cost considerations

To estimate the cost-effectiveness of DNA-informed breeding in perennial crops with multiple years per generation, RosBREED developed and applied a cost estimation tool to examine impacts from technology changes, in our case DNA-informed breeding, on three breeding programs^159–161^, springboarding from a previous RosBREED tool^162^. The decision-support tool, available on the GDR, utilized cost information for the entire breeding process from crossing to cultivar release to provide cost estimates of the effects of new management practices, high-throughput phenotyping, additional procedures, or other variations a breeder might consider for implementation in a program. For the apple breeding program studied^159^, the use of MASS to eliminate greenhouse seedlings prior to field planting was identified as cost-effective when a threshold of at least 25% of the seedlings is discarded. However, for the peach^160^ and strawberry^161^ breeding programs studied, MASS was determined to only be cost-effective at the end of the seedling stage when used to reduce the number of plants advanced to replicated clonal trials. This striking difference was mainly due to the large field cost per seedling in apple compared to peach and strawberry. However, these analyses did not consider goals that can only be accomplished with MASS, such as pyramiding desirable alleles for disease resistance.

#### Apple—Univ. of Minnesota

During RosBREED, the Univ. of Minnesota Apple Breeding Program (UMABP) adopted both MAPS and MASS, exploiting large-effect trait loci contributing to variation for critical fruit quality and disease traits. The UMABP began using DNA information for MAPS in 2012 after finding it was cost-effective. The costs for DNA testing for specific loci or whole-genome DNA profiling of parents were minimal (~$50 per parent) relative to the cost of growing inferior offspring to fruiting (more than $30 per seedling multiplied by hundreds to thousands of seedlings)^159^, highlighting the benefits of MAPS. The trait loci routinely targeted for MAPS include those posing phenotypic evaluation bottlenecks and accounting for a large proportion of culling in the seedling stage. These included disease resistance and fruit quality traits related to texture, taste, storage ability, and fruit appearance, which can only be evaluated when a seedling begins fruiting after several years in the orchard. DNA tests used targeted the trait loci of *MdACS1*, *MdACO1*, *MdPG1*, *Rf*, *Ma*, *Ma3*, and *Rvi6/Vf* (Table 2). The high cost associated with each field-grown apple seedling also resulted in MASS being economically favorable. Therefore, MASS began in the UMABP program in 2014, with one to five loci used for screening each year. From 2017 through 2019, MASS was employed routinely to screen more than 15,600 seedlings, representing 30–70% of all available seedlings and 24–56% of families each year. Culling rates were 35–53% with a cost per culled seedling of $4.59–$7.94, depending on culling rates and the number of DNA tests employed^159^.

The use of GWS for apple seedlings using data from the UMABP was explored in RosBREED by Blissett^163^, examining predictions for fruit quality traits, including acidity, soluble solids content, and texture determined by instrumental and sensory evaluation. The predictive abilities of the models ranged from 0 to 0.52, depending on the genetics of the trait, the population size, and the degree of relationship of the model training population to the test population. Including as few as five full sibs from the test population in the training population provided the ability to predict the performance of other individuals in this family. In apple seedling families, this result suggests that predictions based on the first individuals to fruit in a family were sufficiently predictive of the performance of the remaining family members. In an apple breeding program, where phenotypic evaluations of seedlings are frequently delayed due to biennial fruiting or juvenility, such indirect predictions could be useful to reduce the cost of maintaining these non-phenotyped plant materials^163^.

#### Peach—Clemson Univ

The Clemson Univ. Peach Breeding Program (CUPBP) adopted both MAPS and MASS to reduce the proportion of inferior seedlings in the initial field trials, taking advantage of a few large-effect trait loci. The CUPBP started with two loci for MAPS in 2012 and now uses DNA tests for fruit quality (skin blush, sweetness, fruit size, and *F-M*), productivity (bloom and maturity timing), and disease resistance (*XapF*) (Table 2). Genotypic data from DNA tests, combined with phenotypic data, is now used routinely to design all parental combinations. MASS began in 2016 and is now used routinely prior to field planting to cull seedlings predicted to be susceptible to fruit bacterial spot. Recently, a KASP-based DNA test for bacterial spot fruit response*, PpeXapF*^164^, was developed that was compatible with a crude DNA extraction method and cost only about $1.00 per seedling^125^. Once the peach seedlings were field-planted, newly available DNA tests for fruit quality (skin blush, sweetness, and fruit size) and productivity (bloom and maturity timing) were performed to support decisions about which seedlings to retain in the field and to advance to clonal trials.

GWS was explored for brown rot tolerance because that trait was determined to be controlled by numerous loci with a small effect, for which MASS would not be efficient. Therefore, the feasibility of GWS was evaluated using 26 cultivars and advanced selections and 140 offspring from ten breeding families with the “Bolinha” source of resistance^114^. Predictive ability, assessed over three years as the correlation between observed and predicted phenotypes, was high, ranging from 0.66 to 0.86. As a result, genomic prediction for brown rot was performed in 2020 for use in parental selection and cross design. Due to the high relatedness among peach germplasm across U.S. public breeding programs, CUPBP germplasm will serve as a reference for brown rot resistance prediction in other programs.

#### Sweet cherry—Washington State Univ

The Washington State Univ. breeding program implemented MASS from 2010 for two trait loci. The first selection target was self-compatibility, specifically the self-compatibility allele S_4_’ ^132,165^ (Table 2). The second selection target was a large fruit size based on the fruit size QTL characterized on LG2^165^. For this locus, MASS was most effectively used to select against alleles predictive of small fruit size^136^, although certain alleles and allele combinations were reportedly associated with large fruit size^166^ and selected for in some families. Other trait loci were also RosBREED targets, and DNA-informed breeding was enabled for the *Rf* fruit color locus on LG3^167^ and the maturity timing locus on LG4^168^. The sweet cherry disease resistance focus in RosBREED 2 was powdery mildew, both foliar and fruit. Fortunately, a source of resistance was available and an MTL for both fruit and foliage resistance response was identified on LG5. As with the other valuable trait loci, a DNA test was developed and used in MASS.

#### Sour cherry—Michigan State Univ

MAPS and MASS were first undertaken in the Michigan State Univ. sour cherry breeding program in the mid-2000s for self-compatibility after the self-compatibility alleles were characterized and allele-specific DNA tests developed^131^ (Table 2). In just a few generations, selection using these tests resulted in markedly reduced frequencies of the undesired self-incompatibility alleles in program germplasm. As a result, routine MAPS was performed to monitor the allele prevalence and identify, and therefore consider not making, crosses that would segregate self-incompatible and self-compatible offspring. During RosBREED, the most important use of MASS in sour cherry was for flesh color, addressing the industry’s requirement for bright red fruit^135^. Also being explored for MAPS and MASS in sour cherry is the DNA test for maturity timing developed in sweet cherry, as preliminary investigations indicated that this test could distinguish functional alleles for both bloom and maturity timing in sour cherry.

#### Strawberry—Univ. of Florida

The Univ. of Florida strawberry breeding program (UFSBP) began using MAPS and MASS in 2016, starting with a DNA test for *FAD1* for fruity aroma^169^ and followed by a test targeting *PFRU* for perpetual flowering^128^ (Table 2). DNA tests for newly discovered disease resistance loci were then added for MAPS and MASS, including a test for the *Ca1* locus conferring resistance to fruit and crown rot^121^, *Cg1* for crown rot resistance^122^, *Pc2* for resistance to root and crown rot^123,126^, and *Xf1* for resistance to bacterial angular leaf spot^127^.

These locus-specific DNA tests were highly useful for traits with simple genetics; however, for polygenic traits, GWS was determined to be an effective strategy. In RosBREED, strawberry at the UFSBP was chosen as a model crop/program in which to test GWS due to the ability to apply the method rapidly in multiple cycles. The main findings in strawberry were as follows: (1) markers were more effective than pedigrees for predicting performance, even when phenotypic information was present; (2) breeding values for yield and quality traits could be predicted effectively, with the best parents used in crosses one year early in the cycle^170^; and (3) GWS could be effective for seedling selection as well, to choose the best performing seedlings within families using only 500–700 markers. The conclusion was that if a strawberry breeding program invests in objective measurements of polygenic traits that have at least moderate heritability, then GWS may be a useful strategy for increasing genetic gains for those traits via parental selection, seedling selection, or both.

The UFSBP now uses a combination of both locus-specific DNA tests and GWS for MASS. In one example, locus-specific DNA tests were used to screen ~8000 seedlings for the presence of resistance alleles for *Ca1* and *Pc2*. As these tests were for two unlinked loci, approximately one-fourth of the seedlings (2000) were advanced, which then were subjected to GWS using 500 SNPs. Of these, ~25%, or 500, of the seedlings predicted to have the best performance for fruit sweetness, foliar powdery mildew resistance, and other polygenic traits were advanced to the initial seedling field trials.

### Elite selection advancement

RosBREED 2 conducted pilot studies that directed attention to elite selections in replicated trials—beyond the typical focus of MAS on parents and seedling families. Confidence in the predicted genetic potential of advanced selections is conventionally developed by testing such cultivar candidates as clonal replicates across multiple environments in multi-year trials. Trial environments are planned and assumed to be closely correlated with future commercial production environments. However, such trials are extremely costly, especially in tree crops. RosBREED explored the hypothesis that historical phenotypic data from multiple breeding programs are a sample of the experienced environment and their data sets can be connected through genome-wide multivariate prediction models^171,172^. Such models reduce confounding of prediction of background genetic and QTL variation, thereby improving prediction accuracy in local environments as well as in target untested environments. Large global performance and genotypic data sets were assembled for sweet cherry (762 accessions, 19 locations)^173^, peach (1193 accessions, five locations)^174^, apple (3659 accessions, 19 locations), and strawberry (3368 accessions, eight locations)^175^. Using data for fruit maturity timing in sweet cherry from one location in the U.S. and three locations in Europe, high prediction accuracy (0.85) and genome-by-environment correlations (0.90) were observed^176^. Preliminary analyses in peach using multi-year data from trials at Fresno Calif., College Station, Tex., Clarksville Ark., and Seneca, S.C. demonstrated an increase in prediction accuracy from 0.54, using only local data, to 0.77 when data were combined across locations, and to 0.86 with the inclusion of genotypic categorization by a large-effect QTL (C. Hardner, pers. comm.). In strawberry, opportunities were explored for combining soluble solids content data from temperate and sub-temperate populations on a global scale^175^. The pilot studies demonstrated that this “genomic prediction” approach enables efficient use of existing and future phenotypic and SNP array data for improved germplasm–environment matching, genomic prediction, and QTL characterization. An online tool within the GDR is being developed to enable genomic prediction across a global environmental space^177^.

### Identity and pedigree verification and deduction

RosBREED developed SSR-based fingerprinting tools for blackberry^178^, pear^179^, and cherry rootstock^180^, and shared detailed information about these fingerprinting sets with commercial providers requesting them. Previous fingerprinting assays consisted of multiple PCR reactions and contained di-nucleotide-containing SSRs that are challenging to score due to stuttering, split peaks, and binning errors, leading to genetic profile discrepancies among different laboratories. The newly developed fingerprinting panels for blackberry and pear consists of a single multiplex that amplifies SSR loci with the more reproducible and easier-to-score long core repeats. In addition to confirming parentage of Eastern and Western blackberry breeding populations in RosBREED 2^178^, the blackberry SSR fingerprinting panel proved its use to ascertain parentage of clones and new cultivars^181–183^, including that of the important heritage cultivar Boysen^184^. The newly developed cherry rootstock SSR marker in combination with a previous SSR marker successfully differentiated among commercially available cherry rootstocks^180^. As a result, this SSR pair is used widely in the nursery trade in the U.S. to verify cherry rootstock identity. Parentage records were checked at the onset of RosBREED 1 of plant materials used to create the Crop Reference and Breeding Pedigree Sets of peach, apple, and cherry using a few SSR markers. This verification was to help ensure that the individuals are chosen for expensive phenotyping and genome-wide genotyping came from their supposed parents and would indeed help represent alleles of important breeding parents.

Detailed examinations of identity and pedigrees in U.S. rosaceous crop breeding germplasm were powerfully enabled once SNP array data were obtained. QTL analyses via PBA were strongly supported by the detection of clonal replication, mislabeling, and synonyms as well as by parentage verification or deduction for unselected offspring. Furthermore, pedigrees of parents and ancestors were verified or deduced, culminating in no false pedigree information and numerous new pedigree connections (Fig. 2) that expanded information on shared alleles. This research advance increased statistical power in QTL discovery and characterization and helped reveal sources and inheritance paths of valuable alleles. For example, in sweet cherry, maternity, paternity, or both were deduced or corrected for 86 unselected offspring out of 446 in the germplasm set, leaving only six with one unknown parent^71^. Parentage discoveries in sweet cherry included paternity of the important breeding parents and key ancestors, “Bing”^185^, “Van”, and “Sweetheart”^186^, and many cultivars and selections from the historical WSU breeding program started in the mid-1900s (C. Peace, pers. comm.). Similarly for apple, a multitude of new pedigree connections was made, such as the parentage of the most important U.S. breeding parent, “Honeycrisp”^187^. These many pedigree discoveries were incorporated into the published data set descriptions^71,188^ and QTL studies. Origins of alleles influencing soft scald and fruit acidity alleles in apple were traced back to their earliest known ancestral sources^94,95^. For pre-breeding of apple in RosBREED 2, SNP array data-enabled full parentage deduction of the *BpMADS4* transgenic line and identified the specific ancestral genetic background of the homolog into which the transgene had originally been inserted^155^. The standardized genome-wide data on allelic variation at consistent and known locations along each chromosome for thousands of cultivars, selections, and other individuals of interest has set the stage for deductions of distant pedigree relationships and origin-tracing of valuable alleles. Together with the visualization of the genetic features of elite genomes, this DNA information supports the development of new cultivars targeting ideotypes of desired genomic composition^75,189^.

## A foundation for the future

New knowledge gained of the genome compositions of important crop breeding germplasm, DNA tests for key traits, and genomics tools for further development and application to specific problems will have an ongoing impact on the U.S. and world food system. For rosaceous crops, project outcomes will have lasting utility for breeding programs and provide a roadmap to continue to engage the global research community and deliver superior new cultivars that delight consumers, the rosaceous crop supply chain, and producers alike. In this section, we highlight some additional long-term considerations.

### The legacy of RosBREED

A shared common vision and mission, from stakeholders to scientists, shaped the RosBREED legacy towards its long-term goal: Improved consumption, satisfaction, and sustainability of U.S. rosaceous fruit, nut, and floral crops driven by a continual supply of new disease-resistant cultivars with superior horticultural quality. From the start, the team established the guiding ethos that meaningful impact would only be achieved if genetic discoveries and DNA information were put into practical use within breeding programs to solve high priority stakeholder challenges. Our industry, scientific, and extension stakeholder advisory panels actively participated in project development and execution (Supplementary Tables S1 and S2) and were funded to participate in annual project meetings. Members of our industry stakeholder panel provided critical input on RosBREED activities, communicated directly with their crop-specific organizations, and served as advocates for the RosBREED project specifically, as well as the SCRI program generally. Similarly, members of the extension and scientific advisory panels provided critical input and a portal for extension and outreach beyond rosaceous crops. Their guidance regularly led to a significant adjustment in project direction and activities. Over the 10 years of both RosBREED projects, our project web site, newsletters, workshops, presentations, and peer-reviewed publications were used to share project outputs nationally and internationally. Internally, outreach efforts identified and attempted to engage every rosaceous crop breeding program in the U.S. Newly developed DNA test protocols were shared with project participants, and in many cases site visits by project personnel to individual breeding programs provided hands-on training. Several spin-off projects were generated and a large cohort of scientists trained in developing and implementing modern DNA techniques. In sum, bridging the chasm between ongoing scientific discoveries and practical application required RosBREED to not only discover and validate relevant QTLs, characterize effects of functional alleles, and develop reliable predictive tests, but also to assist breeding programs in broadly implementing the new knowledge and tools, with specific relevance to their local stakeholder priorities, plant materials, program structure, and technical expertise or service providers.

The specific scientific impact of RosBREED on implementation of new knowledge and tools for DNA-informed breeding across U.S. rosaceous crop programs was systematically assessed and quantified during the course of the two projects in four areas of DNA information use:

1. Verifying or understanding germplasm identity or relatedness (including parentage or lineage).
2. Parental selection (assembling and understanding a parent pool, identifying key parents, and/or planning crosses).
3. Seedling screening (selection, sorting, or culling).
4. Upstream research of direct relevance to the program (e.g., QTL analyses using the program’s germplasm, validating DNA tests, or adapting fingerprinting panels) to meet specific program needs.

DNA-informed breeding has become conventional for Rosaceae^14^. By the project’s end in 2019, all RosBREED’s demonstration breeding programs employed DNA information in one or more of the above four areas. More than 75% of these programs reported they now use one or more of these applications on a routine basis, especially for identity/relatedness and parental selection. Further, of 72 additional U.S. rosaceous crop breeding programs, 31% reported ongoing, routine use of DNA information for one or more of these applications (M. Coe, pers. comm.). Together, the RosBREED demonstration and these 72 programs accounted for 92 of the 100 known rosaceous crop breeding programs in the U.S. In sum, as desired by industry stakeholders and as promised by RosBREED, U.S. rosaceous crop breeding programs are implementing DNA information to improve their efficiency and effectiveness in developing new germplasm and delivering superior new cultivars. Furthermore, the training in RosBREED of graduate students and postdoctoral associates (Supplementary Table S3), as well as various staff and undergraduate students, provided individuals to fill breeding and breeding-support positions, and thus filled a void that was identified when designing the first RosBREED project. For example, six DNA-informed breeding graduates of RosBREED are now U.S. fruit breeders.

Although RosBREED activities were funded over a continuous ten-year period, a significant duration for typical USDA grants, ten years is a short time across rosaceous crops to achieve the output of a new cultivar. Nonetheless, some project participants were able to use RosBREED-derived knowledge and strategies to expedite cultivar development and germplasm enhancement within their programs. In apple, the Univ. of Minnesota breeding program utilized DNA testing to predict storage disorders and provide suggested grower/packer storage regimes prior to the 2014 release of “MN55” (now sold as Rave® or First Kiss®)^190^. Similarly, the Univ. of Florida strawberry program applied a DNA test to assure its new release, “Florida Beauty”^191^, carried resistance to the important root rot disease caused by *Phytophthora cactorum*. DNA tests of the nectarine cultivar Amoore Sweet^192^, released in 2013 by the Univ. of Arkansas, confirmed its measured acidity levels and has guided subsequent crosses in the program. Such products are only an initial indicator of the legacy of RosBREED, FruitBreedomics, and other rosaceous crop research projects integrating genomics, genetics, and breeding.

Use of DNA information by rosaceous crop breeders is expected to increase as technologies become more precise and less expensive to apply, and as exciting new target phenotypes are made possible thanks to new genetic findings such as the “jewels in the genome” described above. The students and postdoctoral scientists trained in both projects, along with the many international collaborators (Supplementary Tables S1–S3), another legacy of the two projects, are well-prepared to exploit these advances and deliver superior new cultivars with desirable traits increasingly valued by producers and consumers. The elusive cultivar with excellent fruit quality combined with resistance to diseases prioritized by stakeholders is now within reach, as breeders have better access to parents with pyramided and combined alleles for disease resistance and the tools to track these as well as superior horticultural quality. The ability to select against a “wild” genetic background invigorates disease resistance breeding and simultaneously empowers breeders to seek valuable attributes in previously unused, unadapted germplasm. These superior new cultivars, bred using DNA information, represent a vital RosBREED legacy.

### Future needs

The science of plant breeding will continue to take advantage of advances in -omics biology, including “big data” approaches to phenotypic predictions. High-throughput genotyping and the availability of high-quality, well-curated databases such as the GDR are advancing rapidly in rosaceous crops, but the progress has been slow in high-throughput phenotyping, assisted by robotics, specialized sensors, and “big data” interpretation. Such slow adoption of technological advances is reminiscent of the prior slow adoption of DNA-informed breeding. While the challenges are many, some are simply associated with a lack of needed research and development. For example, no sensors are available to non-destructively, rapidly, and cost-effectively assess the crispness of apple flesh, the juiciness of a peach, or the aroma of a strawberry, nor to provide a reliable assessment of the maturity of any fruit. Furthermore, rosaceous crop breeding programs generally lack sufficient infrastructure support to develop or invest in technological advances. However, as RosBREED demonstrated, these challenges can be met by a collaborative community of industry stakeholders and researchers, dedication to collective advances, and funding of the magnitude necessary to address the scale of the challenges. The global research community is well-positioned for impactful collective advances, thanks in part to the next generation of scientists trained through RosBREED and the relationships that were built or strengthened through the project. Finally, the partnerships strengthened between rosaceous crop breeders and their stakeholders through the RosBREED platform of DNA-informed breeding well-positions these partners to work together to assure the adoption and use of the next generations of breeding technologies.

## Supplementary information

Supplemental material for Iezzoni et al

Table S1. RosBREED 1 project participants and their role and area of expertise, international partners and area of expertise, and Advisory Panel members

Table S2. RosBREED 2 project participants and their role and area of expertise, international partners and area of expertise, and Advisory Panel members

Table S3. RosBREED 1 and 2 post-doctoral associates and graduate students
