# Supplementary material for: RosBREED: bridging the chasm between discovery and application to enable DNA-informed breeding in rosaceous crops
Source: Hortic Res. 2020 Nov 1;7:177. doi: 10.1038/s41438-020-00398-7 (PMC7603521; doi:10.1038/s41438-020-00398-7)
Supplement: Supplementary file 1 — Supplemental material for Iezzoni et al [file 41438_2020_398_MOESM1_ESM.pdf]

Supplementary information for Iezzoni et al., “RosBREED: Bridging the chasm between discovery and application to enable DNA-informed breeding in rosaceous crops”

Three PDF files are included:

Table S1. RosBREED 1 project participants and their role and area of expertise, international partners and area of expertise, and Advisory Panel members.

Table S2. RosBREED 2 project participants and their role and area of expertise, international partners and area of expertise, and Advisory Panel members.

Table S3. RosBREED 1 and 2 post-doctoral associates and graduate students.
