# Supplementary material for: RosBREED: bridging the chasm between discovery and application to enable DNA-informed breeding in rosaceous crops
Source: Hortic Res. 2020 Nov 1;7:177. doi: 10.1038/s41438-020-00398-7 (PMC7603521; doi:10.1038/s41438-020-00398-7)
Supplement: Supplementary file 2 — Table S1. RosBREED 1 project participants and their role and area of expertise, international partners and area of expertise, and Advisory Panel members [file 41438_2020_398_MOESM2_ESM.pdf]

Table S1. RosBREED 1 project participants and their role and area of expertise, international partners and area of expertise, and Advisory Panel members.

Participating Group	Name	Affiliation (during project involvement)	Role/Expertise in RosBREED
**Project team members**			
Project Director (PD)	Amy Iezzoni	Mich. State Univ., East Lansing, Mich.	PD, sour cherry demonstration breeder
Project Co-Director (Co-PD)	Cameron Peace	Wash. State Univ., Pullman, Wash.	Co-PD, Marker-Assisted Breeding Pipeline Team Leader
Co-PIs & Team Leaders	Nahla Bassil	USDA-ARS, Corvallis, Ore.	Genotyping Team Leader
	Gennaro Fazio	USDA-ARS, Geneva, N.Y.	Breeding Information Management System Team Leader
	James Luby	Univ. of Minn., St. Paul, Minn.	Apple Breeding Team Leader, apple demonstration breeder
	Dorrie Main	Wash. State Univ., Pullman, Wash.	Genomics Team Leader
	Cholani Weebadde	Mich. State Univ., East Lansing, Mich.	Extension Team Leader
	Eric van de Weg	Wageningen University and Research, Wageningen, Netherlands	Pedigree-Based Analysis Team Leader
	Chengyan Yue	Univ. of Minn., St. Paul, Minn.	Socio-Economics Team Leader
Co-PIs & Team members	Marco Bink	Plant Research International, Wageningen, Netherlands	Pedigree-Based Analysis
	Susan Brown	Cornell Univ., Geneva, N.Y.	Apple demonstration breeder
	David Byrne	Texas A&M Univ., College Station, Tex.	Peach demonstration breeder
	John R. Clark	Univ. of Arkansas, Fayetteville, Ark.	Peach demonstration breeder
	Carlos Crisosto	Univ. of Calif. – Davis, Davis, Calif.	Peach physiology
	Thomas Davis	Univ. of New Hampshire, Durham, N.H.	Strawberry demonstration breeder
	Kate Evans	Wash. State Univ., Wenatchee, Wash.	Apple demonstration breeder
	Chad Finn	USDA-ARS, Corvallis, Ore.	Strawberry demonstration breeder
	Karina Gallardo	Wash. State Univ., Puyallup, Wash.	Socio-Economics
	Ksenija Gasic	Clemson Univ., Clemson, S.C.	Peach demonstration breeder
	Tom Gradziel	Univ. of Calif. – Davis, Davis, Calif.	Peach demonstration breeder
	James Hancock	Mich. State Univ., East Lansing, Mich.	Strawberry demonstration breeder
	Raymond Jussaume	Wash. State Univ., Pullman, Wash.	Socio-Economics
	Vicki McCracken	Wash. State Univ., Pullman, Wash.	Socio-Economics
	Nnadozie Oraguzie	Wash. State Univ., Prosser, Wash.	Sweet cherry demonstration breeder
	Gregory Reighard	Clemson Univ., Clemson, S.C.	Extension
	Alexandra Stone	Oregon State University, Corvallis, Ore.	Extension
	Dechun Wang	Mich. State Univ., East Lansing, Mich.	Pedigree-Based Analysis
	Kenong Xu	Cornell Univ., Geneva, N.Y.	Extension

Table S1 continued

Participating Group	Name	Affiliation (during project involvement)	Role/Expertise in RosBREED
**Consultant**			
	Michael Coe	Cedar Lake Research Group, Ore.	Extension Evaluation
**International partners**			
	David Chagné	Plant and Food Research, New Zealand	Apple genetics and genomics
	Riccardo Velasco	Istituto Agrario San Michele all'Adige, Trento, Italy	Apple genetics and genomics
	Jasper Rees	Univ. of Western Cape, Bellville, South Africa	Apple genetics and genomics
	Dan Sargent	East Malling Research, East Malling, U.K.	Strawberry genetics and genomics
	Ignazio Verde	Centro di Ricerca per la Frutticoltura, Rome, Italy	Peach genetics and genomics
	Lee Meisel, Herman Silva	Andres Bello Univ., Santiago, Chile	Sweet cherry genetics and genomics
	Francois Laurens, Charles-Eric Durel	Institut National de la Recherche Agronomique, Angers, France	Apple breeding and genetics
	Beatrice Denoyes-Rothan, Elisabeth Dirlewanger, Jose Quero-Garcia	Institut National de la Recherche Agronomique, Bordeaux, France	Strawberry and sweet cherry breeding and genetics
	Benedicte Quilot-Turin, Patrick Lambert, Thierry Pascal	Institut National de la Recherche Agronomique, Avignon, France	Peach breeding and genetics

Table S1 continued

Participating Group	Name	Affiliation
**Advisory Panel members - Industry**		
	Jim Allen	President, New York Apple Association; Board of Trustees, US Apple Assoc.
	Phil Baugher	Co-owner, Adams County Nursery
	Henry Bierlink	Director, Wash. Red Raspberry Commission
	Chalmers Carr III	CEO, Titan Peach Farms, Inc.
	Fred Cook	National Research Dept. Manager, Driscoll's Strawberry Associates
	Robert Curtis	Senior Manager, Almond Board of Calif.
	Bill Dodd	President, Ohio Fruit Growers Marketing Assoc.; Board of Trustees, US Apple Assoc.
	Bruce Grim	Director, Wash. Marketing Assoc.; Executive Director, Wash. State Hortic. Assoc.
	Phil Korson	President, Cherry Marketing Institute
	Jim McFerson	Manager, Washington Tree Fruit Research Commission
	Kevin Moffitt	President, Pears Northwest
	Chrislyn Particka	Research Director, Sakuma Bros., Inc.
	Tom Stokes	CEO, Tree Top Inc.
	Gary van Sickle	Research Director, Calif. Tree Fruit Research Agreement
**Advisory Panel members - Extension**		
	Jessica Goldberger	Wash. State Univ., Pullman, Wash.
	Peter Hirst	Purdue Univ., W. Layfette, Ind.
	David Karp	Univ. of Calif. – Riverside, Riverside, Calif.
	Mercy Olmstead	Univ. of Florida, Gainesville, Fla.
	Ron Perry	Mich. State Univ., E. Lansing, Mich.
	Clark Seavert	Oregon State Univ., Corvallis, Ore.
	Jamie Sherman	Montana State Univ., Bozeman, Mont.
	Brian Sparks	American/Western Fruit Grower
	Chris Watkins	Cornell Univ., Ithaca, N.Y.
**Advisory Panel members - Scientific**		
	Albert Abbott	Clemson Univ., Clemson S.C.
	Pere Arús	Institut de Recerca i Tecnologia Agroalimentàries, Cabrils, Spain
	Joe Arvai	Univ. of Mich., Ann Arbor, Mich.
	Frederick Bliss	Univ. of Calif. – Davis, Davis, Calif. & Seminis Vegetable Seeds, Woodland, Calif.
	Robin Buell	Mich. State Univ., East Lansing, Mich.
	Lailiang Cheng	Cornell Univ., Ithaca, NY
	Susan Gardiner	Plant and Food Research, New Zealand
	Carolyn Ross	Wash. State Univ., Pullman, Wash.
	Phil Simon	USDA-ARS, Madison, Wisc.
