# Supplementary material for: RosBREED: bridging the chasm between discovery and application to enable DNA-informed breeding in rosaceous crops
Source: Hortic Res. 2020 Nov 1;7:177. doi: 10.1038/s41438-020-00398-7 (PMC7603521; doi:10.1038/s41438-020-00398-7)
Supplement: Supplementary file 3 — Table S2. RosBREED 2 project participants and their role and area of expertise, international partners and area of expertise, and Advisory Panel members [file 41438_2020_398_MOESM3_ESM.pdf]

Table S2. RosBREED 2 project participants and their role and area of expertise, international partners and area of expertise, and Advisory Panel members.

Participating Group	Name	Affiliation (during project involvement)	Role/Expertise in RosBREED
**Project team members**			
Project Director (PD)	Amy Iezzoni	Mich. State Univ., East Lansing, Mich.	PD, sour cherry scion and cherry rootstock demonstration breeder
Project Co-Director (Co-PD)	Cameron Peace		Co-PD, DNA-Informed Breeding Team Leader, DNA Testing – apple and cherry
Co-PIs & Team Leaders	Nahla Bassil	USDA-ARS, Corvallis, Ore.	DNA Testing Team Leader, DNA Testing – berry, rose, and pear
	Chad Finn	USDA-ARS, Corvallis, Ore.	Berry Breeding Team Leader, strawberry and blackberry demonstration breeder
	Ksenija Gasic	Clemson Univ., Clemson, S.C.	*Prunus* Breeding Team Leader, peach scion and rootstock demonstration breeder
	Stan Hokanson	Univ. of Minn., St. Paul, Minn.	Rose Breeding Team Leader, Rose demonstration breeder
	James Luby	Univ. of Minn., St. Paul, Minn.	Pome Fruit Team Leader, Apple demonstration breeder
	Dorrie Main	Wash. State Univ., Pullman, Wash.	Data Management Team Leader
	Jim McFerson	Wash. State Univ., Wenatchee, Wash.	Extension Team Leader
	Jay Norelli	USDA-ARS, Kearneysville, WV	Pathology Team Leader, Apple demonstration breeder
	Mercy Olmstead	Univ. of Florida, Gainesville, Fla.	Extension Team Leader
	Vance Whitaker	Univ. of Florida, Wimauma, Fla.	Statistic Genetics Team Leader, Strawberry demonstration breeder
	Chengyan Yue	Univ. of Minn., St. Paul, Minn.	Socio-Economics Team Leader
Co-PIs & Team members	Richard Bell	USDA-ARS, Kearneysville, WV	Pear demonstration breeder
	Rex Bernardo	Univ. of Minn., St. Paul, Minn.	Statistical Genetics
	Marco Bink	Plant Research International, Wageningen, Netherlands	Pedigree-Based Analysis
	Susan Brown	Cornell Univ., Geneva, NY	Apple demonstration breeder
	David Byrne	Texas A&M Univ., College Station, Tex.	Peach demonstration breeder
	John R. Clark	Univ. of Arkansas, Fayetteville, Ark.	Peach demonstration breeder
	Matt Clark	Univ. of Minn., St. Paul, Minn.	Pathology – apple
	Carlos Crisosto	Univ. of Calif. – Davis, Davis, Calif.	Peach standardized phenotyping training
	Thomas Davis	Univ. of New Hampshire, Durham, N.H.	Strawberry demonstration breeder

Table S2 continued

Participating Group	Name	Affiliation (during project involvement)	Role/Expertise in RosBREED
**Project team members cont'd**			
Co-PIs & Team members cont'd	Lisa Wasko DeVetter	Wash. State Univ., Puyallup, Wash.	Extension – small fruit
	Patrick Edger	Mich. State Univ., East Lansing, Mich.	Strawberry genomics and demonstration breeder
	Kate Evans	Wash. State Univ., Wenatchee, Wash.	Apple demonstration breeder
	Karina Gallardo	Wash. State Univ., Puyallup, Wash.	Socio-Economics (interim Socio-Economics Team Leader)
	Tom Gradziel	Univ. of Calif. – Davis, Davis, Calif.	Peach demonstration breeder
	James Hancock	Mich. State Univ., East Lansing, Mich.	Strawberry demonstration breeder
	Craig Hardner	Univ. of Queensland, Australia	Statistical Genetics
	Kelly Ivors	Calif. Poly. State Univ., San Luis Obispo, Calif.	Pathology – strawberry
	Amy Lawton-Rauh	Clemson Univ., Clemson SC	Statistical Genetics, genome evolution
	Desmond Layne	Wash. State Univ., Pullman, Wash	Extension
	Lise Mahoney	Univ. of New Hampshire, Durham, N.H.	Strawberry demonstration breeder
	Per McCord	Wash. State Univ., Prosser, Wash.	Sweet cherry demonstration breeder
	Vicki McCracken	Wash. State Univ., Pullman, Wash.	Socio-Economics
	Nnadozie Oraguzie	Wash. State Univ., Prosser, Wash.	Sweet cherry demonstration breeder
	Gregory Reighard	Clemson Univ., Clemson, S.C.	Extension – tree fruit
	Chris Saski	Clemson Univ., Clemson, S.C	DNA Testing – *Prunus*
	Guido Schnabel	Clemson Univ., Clemson, S.C	Pathology
	Eric van de Weg	Wageningen Univ. and Research, Wageningen, Netherlands	Pedigree-Based Analysis
**Breeder collaborators**			
	Bill Shane	Mich. State Univ., Benton Harbor, Mich.	Peach breeder
	Patrick Moore	Wash. State Univ., Puyallup, Wash.	Strawberry breeder
**Consultant**			
	Michael Coe	Cedar Lake Research Group, Ore.	Extension Evaluation

Table S2 continued

Participating Group	Name	Affiliation (during project involvement)	Role/Expertise in RosBREED
**International partners**			
	David Chagné	Plant and Food Research, New Zealand	Apple genetics and genomics
	Riccardo Velasco	Istituto Agrario San Michele all'Adige, Trento, Italy	Apple genetics and genomics
	Jasper Rees	Univ. of Western Cape, Bellville, South Africa	Apple genetics and genomics
	Richard Harrison, Felicidad Fernandez	East Malling Research, East Malling, U.K.	Strawberry genetics and genomics; cherry and pear breeding and genetics
	Ignazio Verde	Centro di Ricerca per la Frutticoltura, Rome, Italy	Peach genetics and genomics
	Lee Meisel, Herman Silva	Univ. of Chile., Santiago, Chile	Peach, strawberry, sweet cherry genetics and genomics
	Francois Laurens, Charles-Eric Durel	Institut National de la Recherche Agronomique, Angers, France	Apple breeding and genetics
	Beatrice Denoyes-Rothan, Elisabeth Dirlewanger, Jose Quero-Garcia	Institut National de la Recherche Agronomique, Bordeaux, France	Strawberry and sweet cherry breeding and genetics
	Benedicte Quilot-Turin, Patrick Lambert, Thierry Pascal	Institut National de la Recherche Agronomique, Avignon, France	Peach breeding and genetics
	Thomas Debener	Leibniz Univ., Hannover, Germany	Rose genetics and genomics
	Pere Arús, Amparo Monfort	Institute de Recerca i Tecnologia Agroalimentàries, Cabrils, Spain	Peach and strawberry genetics and genomics
	Iraida Amaya	Instituto de Investigación y Formación Agraria y Pesquera, Málaga, Spain	Strawberry breeding, genetics, and genomics

Table S2 continued

Participating Group	Name	Affiliation
**Advisory Panel members - Industry**		
	Jim Allen	President, New York Apple Association; Board of Trustees, US Apple Assoc.
	Jen Baugher	Marketing Associate, Adams County Nursery
	Chalmers Carr III	CEO, Titan Peach Farms, Inc.
	Robert Curtis	Director, Agricultural Affairs, Almond Board of California
	Bill Dodd	President, Ohio Fruit Growers Marketing Assoc.; Board of Trustees, US Apple Assoc.
	Carlos Fear	Director of Consumer Lab and Molecular Genetics, Driscoll's Strawberry Associates
	Bob Gix	Horticulturalist, Blue Star Growers
	Bruce Grim	Director, Wash. Marketing Assoc.; Executive Director, Wash. State Hortic. Society
	Cynthia Haskins	President and CEO, New York Apple Assoc.
	Phil Korson	President, Cherry Marketing Institute
	Dan Legard	Director, Calif. Strawberry Commission
	John Lott	President, Bear Mountain Orchards, Aspers, Penn.
	Gabrielle Ludwig	Director, Sustainability & Environmental Affairs, Almond Board of Calif.
	Mercy Olmstead	Senior Manager, Production Research & Education Calif. Strawberry Commission
	Chris Pellett	Co-owner, NewFlora LLC, Central Point, Ore.
**Advisory Panel members - Extension**		
	David Eddy	Editor, American/Western Fruit Grower
	Peter Hirst	Purdue Univ. West Lafayette, Ind.
	David Karp	Univ. of Calif. – Riverside, Riverside, Calif.
	Ron Perry	Mich. State Univ., East Lansing, Mich.
	Clark Seavert	Oregon State Univ., Corvallis, Ore.
	Chris Watkins	Cornell Univ., Ithaca, N.Y.
**Advisory Panel members - Scientific**		
	Pere Arús	Institut de Recerca i Tecnologia Agroalimentàries, Cabrils, Spain
	Joe Arvai	Univ. of Mich., Ann Arbor, Mich.
	Frederick Bliss	Univ. of Calif. – Davis, Davis, Calif. & Seminis Vegetable Seeds, Woodland, Calif.
	Robin Buell	Mich. State Univ., East Lansing, Mich.
	Lailiang Cheng	Cornell Univ., Ithaca, N.Y.
	Susan Gardiner	Plant and Food Research, New Zealand
	Kim Hummer	USDA-ARS, Corvallis, Ore.
	Dan Kluepfel	Univ. of Calif. – Davis, Davis, Calif.
	Brad Rickard	Cornell Univ., Ithaca, N.Y.
	Carolyn Ross	Wash. State Univ., Pullman, Wash.
	Phil Simon	USDA-ARS, Madison, Wisc.
