# Supplementary material for: RosBREED: bridging the chasm between discovery and application to enable DNA-informed breeding in rosaceous crops
Source: Hortic Res. 2020 Nov 1;7:177. doi: 10.1038/s41438-020-00398-7 (PMC7603521; doi:10.1038/s41438-020-00398-7)
Supplement: Supplementary file 4 — Table S3. RosBREED 1 and 2 post-doctoral associates and graduate students [file 41438_2020_398_MOESM4_ESM.pdf]

Table S3. RosBREED 1 and 2 post-doctoral associates and graduate students.

Name	RosBREED Advisor(s)	Institution(s)
**Post-doctoral associates**		
Laima Antanaviciute	Ksenija Gasic	Clemson Univ.
Lichun Cai	Amy Iezzoni	Mich. State Univ.
Cassia Da Silva Linge	Ksenija Gasic	Clemson Univ.
Mulusew Fikere	Craig Hardner	University of Queensland
Jerry Hill	Dorrie Main, Cameron Peace	Wash. State Univ.
Lise Mahoney	Tom Davis	Univ. of New Hamp.
Youngjae Oh	Vance Whitaker	Univ. of Fla.
Julia Piaskowski	Cameron Peace	Wash. State Univ.
Umesh Rosyara	Amy Iezzoni	Mich. State Univ.
Sushan Ru	Dorrie Main	Wash. State Univ.
Stijn Vanderzande	Cameron Peace	Wash. State Univ.
Sujeet Verma	Vance Whitaker	Univ. of Fla
Nan Yang	R. Karina Gallardo, Vicki McCracken	Wash. State Univ.
Ping Zheng	Dorrie Main	Wash. State Univ.
Laura Ziems	Craig Hardner	University of Queensland
Jason Zurn	Nahla Bassil	USDA-ARS, Corvallis
**Graduate students**		
Kristen Andersen	Amy Iezzoni	Mich. State Univ.
Nita Basundari	Amy Iezzoni	Mich. State Univ.
Elisabeth Blissett	Rex Bernardo, Jim Luby	Univ. of Minn.
Jong Choi	Chengyan Yue	Univ. of Minn.
Matt Clark	Jim Luby	Univ. of Minn.
Jonathan Fresnedo-Ramirez	Tom Gradziel	Univ. of Calif. – Davis
Terrence Frett	Ksenija Gasic / John Clark	Clemson Univ. / Univ. of Ark.
Wanfang Fu	Ksenija Gasic	Clemson Univ.
Yingzhu Guan	Kate Evans	Wash. State Univ.
Julia Harshman	Kate Evans	Wash. State Univ.
Nicholas Howard	Jim Luby	Univ. of Minn.
Tymon James	Cameron Peace	Wash. State Univ.
Alexandra Johnson	Cameron Peace	Wash. State Univ.
Stella Kang	David Byrne	Texas A&M Univ.
Sarah Kostick	Kate Evans	Wash. State Univ.
Huixin Li	Vicki McCracken, R. Karina Gallardo	Wash. State Univ.
Yingzi Li	Vicki McCracken, R. Karina Gallardo	Wash. State Univ.
Zongyu Li	Vicki McCracken, R. Karina Gallardo	Wash. State Univ.
Lise Mahoney	Tom Davis	Univ. of New Hamp.
Ben Orcheski	Susan Brown	Cornell Univ.
Zena Rawandoozi	David Byrne	Texas A&M Univ.
Kathleen Rhoades	Amy Iezzoni	Mich. State Univ.
Lilian Carrillo Rodriguez	Vicki McCracken, R. Karina Gallardo	Wash. State Univ.
Sushan Ru	Dorrie Main, Cameron Peace	Wash. State Univ.
Alejandra Salgado	John Clark	Univ. of Ark.

Table S3 continued

Name	RosBREED Advisor(s)	Institution(s)
Natalia Salinas	Nahla Bassil / Vance Whitaker	USDA-ARS, Corvallis / Univ. of Fla.
Paul Sandefur	John Clark / Cameron Peace	Univ. of Ark. / Wash. State Univ.
Alexander Schaller	Cameron Peace	Wash. State Univ.
Cari Schmitz-Carley	Jim Luby	Univ. of Minn.
Travis Stegmeir	Amy Iezzoni	Mich. State Univ.
John Tillman	Jim Luby	Univ. of Minn.
Sujeet Verma	Cameron Peace	Wash. State Univ.
Jingjing Wang	Chengyan Yue	Univ. of Minn.
Seth Wannemuehler	Chengyan Yue	Univ. of Minn.
Muqing Yan	Dave Byrne	Texas A&M Univ.
Melinda Yin	John Clark, Margaret Worthington	Univ. of Ark.
Ellen Young	David Byrne	Texas A&M Univ.
Shuoli Zhao	Chengyan Yue	Univ. of Minn.
Yunyang Zhao	Nnadozie Oraguzie	Wash. State Univ.
Quijie Zheng	Vicki McCracken	Wash. State Univ.
Ruchen Zhou	Chengyan Yue	Univ. of Minn.
